# In Silico and In Vitro Studies of Terpenes from the Fabaceae Family Using the Phenotypic Screening Model against the SARS-CoV-2 Virus

**DOI:** 10.3390/pharmaceutics16070912

**Published:** 2024-07-09

**Authors:** Natália Ferreira de Sousa, Gabrielly Diniz Duarte, Carolina Borsoi Moraes, Cecília Gomes Barbosa, Holli-Joi Martin, Nail N. Muratov, Yuri Mangueira do Nascimento, Luciana Scotti, Lúcio Holanda Gondim de Freitas-Júnior, José Maria Barbosa Filho, Marcus Tullius Scotti

**Affiliations:** 1 Postgraduate Program in Natural and Synthetic Bioactive Products, Federal University of Paraíba, João Pessoa 58051-900, Brazil; nataliafsousa@ltf.ufpb.br (N.F.d.S.); yurimangueira@ltf.ufpb.br (Y.M.d.N.); luciana.scotti@gmail.com (L.S.); barbosa.ufpb@gmail.com (J.M.B.F.); 2 Postgraduate Program in Development and Innovation of Drugs and Medicines, Federal University of Paraíba, João Pessoa 58051-900, Brazil; gabriellydduarte@gmail.com; 3 Institute of Biomedical Sciences, University of São Paulo (ICB-USP), São Paulo 05508-000, Brazil; cbmoraes@unifesp.br (C.B.M.); cecigomes.barbosa@gmail.com (C.G.B.); luciofreitasjunior@gmail.com (L.H.G.d.F.-J.); 4 Eshelman School of Pharmacy, University of North Carolina, Chapel Hill, NC 27599, USA; holli27@unc.edu; 5 Department of Chemical Technology, Odessa National Polytechnic University, 65000 Odessa, Ukraine; nail_muratov@ukr.net; 6 A. V. Bogatsky Physical-Chemical Institute of NASU, 65047 Odessa, Ukraine

**Keywords:** virtual screening, SARS-CoV-2, natural products, phenotypic screening, Fabaceae family

## Abstract

In 2019, the emergence of the seventh known coronavirus to cause severe illness in humans triggered a global effort towards the development of new drugs and vaccines for the SARS-CoV-2 virus. These efforts are still ongoing in 2024, including the present work where we conducted a ligand-based virtual screening of terpenes with potential anti-SARS-CoV-2 activity. We constructed a Quantitative Structure–Activity Relationship (QSAR) model from compounds with known activity against SARS-CoV-2 with a model accuracy of 0.71. We utilized this model to predict the activity of a series of 217 terpenes isolated from the Fabaceae family. Four compounds, predominantly triterpenoids from the lupane series, were subjected to an in vitro phenotypic screening in Vero CCL-81 cells to assess their inhibitory activity against SARS-CoV-2. The compounds which showed high rates of SARS-CoV-2 inhibition along with substantial cell viability underwent molecular docking at the SARS-CoV-2 main protease, papain-like protease, spike protein and RNA-dependent RNA polymerase. Overall, virtual screening through our QSAR model successfully identified compounds with the highest probability of activity, as validated using the in vitro study. This confirms the potential of the identified triterpenoids as promising candidates for anti-SARS-CoV-2 therapeutics.

## 1. Introduction

The Coronavirus Disease 2019 (COVID-19) pandemic has had a profound global impact on global human health, with over 774 million confirmed cases and over 7 million deaths worldwide as of March 2024 [1]. This viral disease demonstrates a wide range of symptoms, from mild to severe pneumonia and acute respiratory distress, to a multi-organ disorder affecting various systems, including the pulmonary, cardiovascular, neurologic, renal, endocrine, dermatologic, and gastrointestinal systems [2]. The virus has undergone continuous evolutionary adaptations since its emergence which impact its interactions with our biological systems, enabling the virus to bypass immune defenses while diminishing its virulence. This evolution has been observed in variants such as Omicron BA.1 and others, with extensive sequencing and data analysis conducted over the past four years [3,4,5].

Since its initial emergence in Wuhan, China, in 2019, the management of COVID-19 has significantly changed, transitioning from early strategies such as social distancing and lockdowns to more advanced approaches, including intensive care, vaccination campaigns, and the development of antiviral drugs and monoclonal antibodies [6]. The SARS-CoV-2 virus, responsible for COVID-19, comprises a structural spike protein which is pivotal in its binding with host cell receptors to initiate the viral life cycle [7]. Remarkably, the virus can mutate its spike proteins to evade host defenses, presenting challenges for vaccinated individuals and contributing to recurrent infections [8]. Conversely, in anti-SARS-CoV-2 drug discovery, many studies have concentrated on attacking SARS-CoV-2 with small molecule inhibitors that block viral proteases and polymerases, including RNA-dependent RNA polymerase (RdRp) [9], the main protease (Mpro or 3CLpro), and the papain-like protease (PLpro) [10], which are instrumental in the development of novel compounds [11]. One popular method for testing the in vitro activity of these compounds is phenotypic screening based on the activation of caspase 3/7 in Vero cells [12,13].

In addition to the development of pharmaceutical drugs and monoclonal antibodies, reports have surfaced from Zimbabwe [14], Nigeria [15], and India [16] that suggest the efficacy of medicinal plants in managing COVID-19 symptoms. These studies often involve the utilization of crude plant extracts or purified compounds from the plant families Fabaceae and Lamiaceae [17]. The Fabaceae family is considered the second-most diverse and economically important plant family, and includes several medicinally significant plants known for their antimicrobial, anticancer, antibacterial, diuretic, and anti-inflammatory properties such as *Melilotus officinalis* (MO), *Coronilla varia* (CV), *Ononis spinosa* (OS), and *Robinia pseudoacacia* (RP) [18,19,20,21,22,23]. These are particularly intriguing because they contain an abundance of secondary metabolites, some of which have demonstrated pharmacological activity in vitro [24]. Among these compounds are terpenes, a class of natural volatile compounds with more than 80,000 screened for potential therapeutic applications [25], including antiviral activity against various Human Coronaviruses (HCoVs) [26].

This study aims to explore the anti-SARS-CoV-2 potential of terpenes isolated from the Fabaceae family. To do this, we created an in silico Quantitative Structure–Activity Relationship (QSAR) model from compounds with known activity against SARS-CoV-2. We utilized this model to predict the activity of a series of 217 terpenes isolated from the Fabaceae family and subjected promising compounds to an in vitro phenotypic screening in Vero CCL-81 cells to assess their inhibitory activity against SARS-CoV-2. The compounds which showed high rates of SARS-CoV-2 inhibition along with substantial cell viability underwent molecular docking at the SARS-CoV-2 main protease, papain-like protease, spike protein and RNA-dependent RNA polymerase.

## 2. Materials and Methods

### 2.1. Extraction of Compounds the Study

Betulinic acid was extracted from the bark of *Zizhyphus joazeiro* Mart. (Rhamnaceae) using a method previously described by Barbosa Filho and collaborators in 1985 [27,28]. Column chromatography was employed for the extraction and acid hydrolysis was performed. The isolation process involved comparing physical properties obtained through various spectrometric methods (Infrared Spectrometry (IV), Ultraviolet Spectrometry (UV), Mass Spectrometry (MS), Hydrogen Nuclear Resonance Spectrometry (H-NMR) and Carbon Nuclear Resonance Spectrometry (C-NMR)).

Lupeol was extracted from the bark of *Lonchocarpus araripensis* Benth. (Fabaceae) using a method previously outlined [29,30] by Barbosa and collaborators (2013). The fractions corresponding to the crude ethanolic extract were monitored using Analytical Thin Layer Chromatography, with lupeol being identified using nuclear magnetic resonance spectroscopic data and through a comparison with values reported in the literature. The substances betulinic acid acetate and betulinic acid methyl ester were purchased from the company Sigma Aldrich, St. Louis, MO, USA (https://www.sigmaaldrich.com/BR/pt accessed on 14 January 2024).

### 2.2. Data Collection and Curation

The CHEMBL database (EMBL-EBI, Wellcome Genome Campus, Hinxton, UK; https://www.ebi.ac.uk/chembl/ accessed on 28 October 2023) was used to extract 412 compounds with reported in vitro anti-SARS-CoV-2 activity (pIC_50_) (ChEMBL ID: 4,303,835—organism) in Vero E6 cells, Vero C1008 Cells, A549-ACE2, Caco-2, Huh-7, and Calu-3 in various assays. We used a binary classification system where compounds with reported pIC50 ≥ 5.9355 were considered active (105 compounds), while compounds with pIC50 ≤ 5.8894 were classified as inactive (305 compounds). We used a standard 80–20 split for the training to test set, where 330 compounds constituted the training series, and 82 compounds formed the test series. We used a 10-fold cross-validation for our training set, meaning that 10% of the training series (33 compounds) was left out for internal validation and this was repeated 10 times, with a new set of 33 compounds used for training each iteration. The cross-validation was employed using a stratified approach, ensuring that the proportion of active and inactive compounds was maintained during the removal process. We used the external test set of 82 compounds (21 active compounds and 61 inactive compounds) to validate our model and generate our models statistics.

To obtain our prediction set, we queried the Web of Science database (https://www.webofscience.com/wos/woscc/basic-search accessed on 31 March 2022) using the keywords “Fabaceae”, “terpene” and “*Leguminosae”*. We collected data from 77 articles published between 1991 and 2023 and identified 217 compounds derived from natural products belonging to the class of terpenes found in the Fabaceae family (*Leguminosae*) (Table S1—Supplementary Materials). We cross referenced this list of compounds with those used to develop our QSAR model to ensure there was no overlapping compounds.

The compounds chemical structures were designed using the Marvin Sketch 18.14 software program 2017 by ChemAxon (https://chemaxon.com/ accessed on 14 October 2023), and subsequently converted into SMILES. We used Chemaxon Standardizer v.18.17.0 (ChemAxon, Boston, MA, USA, www.chemaxon.com accessed on 14 October 2023) to transform the chemical structures into a uniform representation to avoid inconsistencies. This process included the addition of explicit hydrogen atoms, neutralizing charged fragments or functional groups, recognizing and converting legacy representations of functional groups (like aliases), removing water and salt counterions, expanding abbreviated groups, and a conversion to 3D representation. The tool also ensures a unified representation of aromatic rings, tautomers and mesomers [31,32]. We compiled these compounds in a database and integrated it into the Sistemat X Web platform (https://sistematx.ufpb.br/ accessed on 14 October 2023) (Table S1—Supplementary Materials).

### 2.3. QSAR Modeling

The Knime 3.6.2 software (Knime 3.6.2, Copyright Miner, de Konstanz Information, Zurich, Suíça, www.knime.com accessed on 14 October 2023) was employed to build and evaluate the QSAR models. Given the success of previous studies conducted by our group [33,34], we utilized 3D QSAR analysis. To accomplish this, all compounds were converted into 3D structures, saved in SDF format, and then imported into AlvaDesc descriptors (https://www.alvascience.com/alvadesc/ accessed on 14 October 2023) [35,36] to obtain the necessary descriptors.

The Random Forest (RF) algorithm was selected to build the predictive model. The applicability domain was calculated based on the Euclidean distances present in the surveyed chemical space [34]. External cross-validation was conducted to estimate the predictive power of the developed model. The performance of the external predictions was assessed through Area Over the Curve analysis (ROC). Additionally, the models underwent analysis using the Matthews Correlation Coefficient (MCC) confusion matrix to provide a comprehensive evaluation of the model’s effectiveness.

### 2.4. Biological Assays

#### 2.4.1. Cell Line

Vero cells (CCIAL 057) were obtained from the “Núcleo de Cultura de Células—Instituto Adolfo Lutz, São Paulo, Brazil”. The cells were cultured in high glucose DMEM medium (Sigma-Aldrich), supplemented with 10% heat-inactivated fetal bovine serum (FBS) (Thermo Scientific, Waltham, MA, USA) and 100 U mL^−1^ Streptomycin (Thermo Scientific) at 37 °C with 5% CO_2_.

#### 2.4.2. Virus Strain

All procedures involving the SARS-CoV-2 virus were performed in the level 3 biosafety laboratory of the Institute of Biomedical Sciences of the University of São Paulo. The SARS-CoV-2 virus used in this study (HIAE-02: SARS-CoV-2/SP02/human/2020/ARB, GenBank Accession No. MTI26808.1) was isolated from a nosopharyngeal sample of a confirmed COVID-19 patient at Hospital Israelita Albert Einstein, São Paulo (SP) Brazil.

#### 2.4.3. Phenotypic Screening with SARS-CoV-2

The test compounds were initially diluted to a concentration of 2 mg/mL in Dimethyl sulfoxide (DMSO). Subsequently, these compounds were tested at a single concentration of 10 μg/mL. Before treating the cells, the compounds underwent a 33.33x dilution in Phosphate-buffered saline (PBS), and 10 µL of each dilution was transferred to the assay plates, resulting in a final dilution factor of 200×. The tests were conducted in duplicate, with chloroquine utilized as a control [13].

For the phenotypic screening of compounds, 2000 Vero cells were seeded per well in 384-well plates in Dulbecco’s Modified Eagle’s Medium (DMEM) supplemented with 10% heat-inactivated fetal bovine serum (Thermo Scientific). The cells were incubated at 37 °C with 5% CO_2_. After 24 h, the cells were treated with the compounds as described above, followed by the addition of the virus at a Multiplicity of Infection (MOI) of 0.1 viral particles per cell. The final concentration of DMSO in the assay plates was 0.5% (*v*/*v*). After 33 h, the plates were fixed, immunofluorescence was performed using serum from COVID-19 patients, and the images were acquired and analyzed using the Operetta High-Content Analysis System (HCS) equipment [13].

The images were subjected to an automated detection of infected and non-infected cells. The parameters measured in each well included the total number of cells and total number of infected cells. Infection was quantified as the percentage of infected cells relative to the total number of cells. The reduction in the number of infected cells reflected the percentage of antiviral activity exhibited by the samples. The activity of each compound was normalized against infected and uninfected controls, as was the cell survival rate. The cell survival rate is expressed as the percentage of the number of cells in the test well in relation to the average number of cells in the infected control wells [13].

The EC_50_ was defined as the compound concentration causing a 50% reduction in viral infection compared to infected controls. The CC_50_ value was defined as the compound concentration causing a 50% reduction in cell survival compared to infected controls. The Selectivity Index was calculated as the ratio between CC_50_ and EC_50_ (CC_50_/EC_50_). Maximum Activity represented the maximum inhibition of infection observed compared to controls.

### 2.5. Molecular Docking Studies

Proteins were downloaded from the Protein Data Bank (PDB) library (https://www.rcsb.com/ accessed on 14 October 2023) [37]. The targets were selected through bibliographical research concerning the mechanism of action involved in the inhibition of the SARS-CoV-2, taking into account their structural similarity. The obtained structures were as follows: main protease (M-pro) in complex with NCL-00024905 (PDB: 5RG1), resolution: 1.65 Å and method: X-Ray Diffraction [38]; papain-like protease (PL-pro) in complex with inhibitor 3k (PDB: 7TZJ), resolution: 2.66 Å and method: X-Ray Diffraction [39]; spike glycoprotein in complex with the 10D12 heavy-chain-only antibody (local refinement) (PDB: 8C8P), resolution: 4.10 Å and method: Electron microscopy; and SARS-CoV-2 RNA-dependent RNA polymerase in complex with cofactors (PDB: 6M71) [40], resolution: 2.9 Å.

The active binding sites of the proteins were determined based on a literature search and were included in the docking study [41]. The active site was defined based on the active site information available in the referenced articles. For the proteins, M-protease (PDB: 5RG1) and PL-protease (PDB: 7TZJ), which had co-crystallized ligands, the active site was defined through the template established through the coordinates of the ligand in contact with the protein. Regarding the spike glycoprotein (PDB: 8C8P) and the RNA-dependent RNA polymerase (PDB: 6M71), which did not have co-crystallized ligands, the active site was determined using molecular pocket predictions from the platform Bite Net—Skolteck I Molecule, 2023 (https://sites.skoltech.ru/imolecule/tools/bitenet accessed on 14 October 2023). These predictions indicated that the active site comprises the region equivalent to the terminal end of the A subunit. Furthermore, for the spike protein, the drug Nirmatrelvir was employed as a positive control [42], and for the RNA-dependent RNA polymerase, the drug Remdesivir was employed as a positive control [40].

Redocking was conducted as a preliminary step to validate the docking simulation. Both procedures were performed using Molegro Virtual Docker (MVD) v.6.0.1 software [43]. Enzymes and compounds were prepared according to predefined parameters within the software.

In the coupling procedure (linker–enzyme), a grid of 15 Å radius and a resolution of 0.30 was utilized. This grid encompassed the binding site, as defined by a known ligand for each enzyme. A model was generated to perform and evaluate the fit with expected characteristics between the ligand and the enzyme, using the MOLDOCK Score (GRID) algorithm with the scoring function and search algorithm, corresponding to Moldock. The MolDock scoring function enhances these scoring functions with a new hydrogen bonding term and new charge schemes. The docking scoring function, Escore, is defined by the following energy terms:

Escore = Einter + Eintra

The visualization of the established interactions was performed in the Discovery Studio Visualizer program, Biovia, 2021 v21 1.0 (https://www.3dsbiovia.com/, https://sistematx.ufpb.br/ accessed on 14 October 2023) [44].

## 3. Results

### 3.1. Compounds in Study

The study comprises 217 natural products classified as terpenes, including sesquiterpenes, diterpenes, monoterpenes, and triterpenes occurring in the Fabaceae family (*Leguminosae*). These compounds were identified through an exhaustive literature search conducted on the Web of Sciences database (https://www.webofscience.com/wos/woscc/basic-search accessed on 31 March 2022). Upon compiling the database, it was deposited on the Sistemat X web platform (https://sistematx.ufpb.br/ accessed on 14 October 2023). For a detailed description of the compounds under investigation obtained through the literature review, please refer to Table S1 of the Supplementary Materials.

### 3.2. Quantitative Structure–Activity Relationship (QSAR) Modeling

A classification model was developed for ligand-based virtual screening, employing the Random Forest (RF) algorithm. The physicochemical properties of the compounds were determined by AlvaDesc descriptors (https://www.alvascience.com/alvadesc/ accessed on 14 October 2023) [35,36]. The developed model underwent validation, and its predictive capacity was assessed using parameters such as specificity, sensitivity, accuracy and precision. The performance and robustness of the models were appraised through the Receiver Operating Characteristic (ROC) Curve. Table 1 provides detailed information on the parameters of the model created with the AlvaDesc descriptors.

Our model underwent cross-validation that affirmed good performance results with accuracy values exceeding 70%. The ROC Curve values for the developed model were greater than 0.80, signifying a robust and predictive model. The model demonstrated high specificity, with values of 0.75 (Test) and 0.702 (Cross). Similarly, high and satisfactory values for sensitivity were observed, as these corresponded to 0.759 (Test) and 0.716 (Cross). In general, it was observed that the model presents a good prediction as it presented Matthews Correlation Coefficient (MCC) values corresponding to 0.5 (Test) and 0.431 (Cross).

The model built on AlvaDesc descriptors for SARS-CoV-2 (Table S2—Supplementary Materials) predicted the compounds within the applicability domain, with the exception of 119 compounds (1–17, 19, 24–25, 27, 30–34, 36–51, 53–59, 62, 64, 66–70, 72–75, 80, 82–102, 105–118, 130, 133–134, 139–142, 144–146, 148–151, 163–165, 175–176, 186, 188–189, 213, and 214) (Table S2 and Figure S1—Supplementary Materials). Furthermore, the model classified 85 compounds with a probability of activity above 50% and the applicability domain as being reliable, with probability values corresponding to 0.50 to 0.589 (Table S3 and Figure S2—Supplementary Materials). These selected compounds corresponded to 168, 204, and 2015 (*p* = 0.589); 123, 172, 194, 198, 206, 210, and 211 (*p* = 0.579); 76, 77, 120, 138, 157, 162, 170, 171, 178, 195, 196, and 212 (*p* = 0.569); 28, 52, 60, 78, 119, 135, 136, 158, 160, 169, 192, 193, and 197 (*p* = 0.560); 104, 121, 128, 132, 153, 154, 173, 180, 181, 182, 199, and 209 (*p* = 0.550); 29, 63, 122, 129, 131, 147, 155, 161, 167, 205, 207, and 208 (*p* = 0.540); 126, 127, 137, 156, 174, 183, 184, 187, 202, 216, and 217 (*p* = 0.529); 124, 125, 166, 179, 185, 200, and 203 (*p* = 0.519) and 103, 143, 159, 190, 191, 65, 152, and 201 (*p* = 0.509); 65, 152, and 201 (*p* = 0.5). The chosen compounds represented the classes of diterpenes, rare monoterpenes substituted with osidic units, cumaric groups, and triterpenes. Figure S2 (Supplementary Materials) illustrates the chemical structure of the compounds that exhibited activity probability values above 0.5 and a reliable applicability domain in the prediction model created with AlvaDesc descriptors.

### 3.3. Selection of Molecules for Biological Test

**Figure 1:**
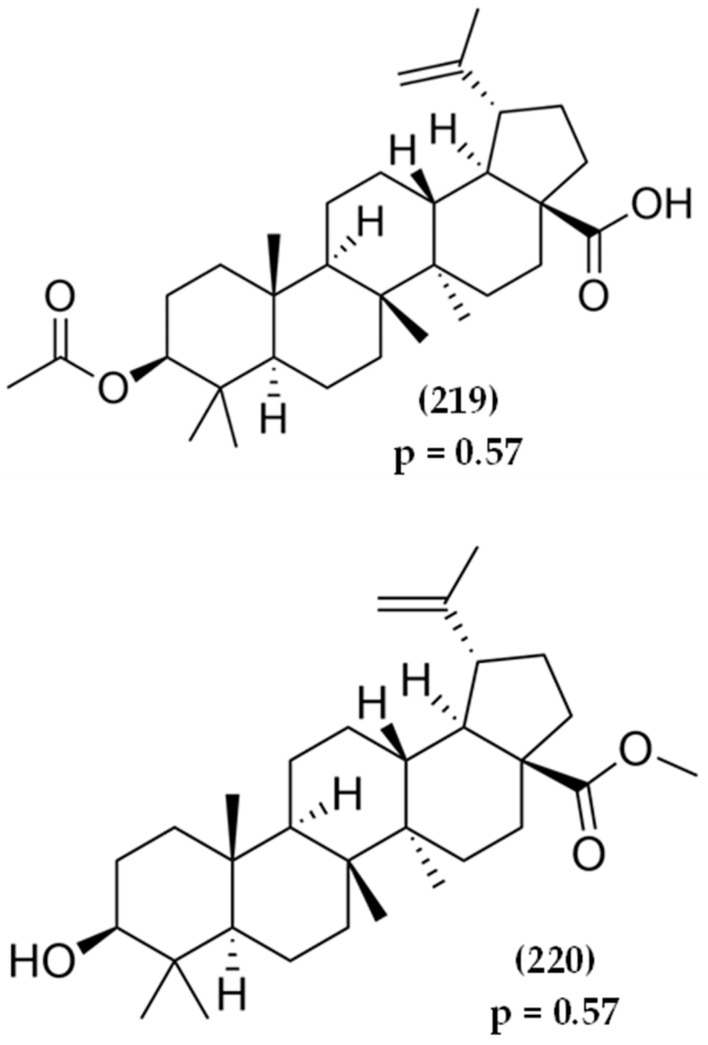
Structure of the synthetic compounds derived from the triterpenes under study.

After conducting in silico screening, 86 compounds were chosen for the evaluation of in vitro biological activity, namely: 168, 204, and 215 (*p* = 0.589); 123, 172, 194, 198, 206, 210, and 211 (*p* = 0.579); 76, 77, 120, 138, 157, 162, 170, 171, 178, 195, 196, and 212 (*p* = 0.569); 28, 52, 60, 78, 119, 135, 136, 158, 160, 169, 192, 193, and 197 (*p* = 0.560); 104, 121, 128, 132, 153, 154, 173, 180, 181, 182, 199, and 209 (*p* = 0.550); 29, 63, 122, 129, 131, 147, 155, 161, 167, 205, 207, and 208 (*p* = 0.540); 126, 127, 137, 156, 174, 183, 184, 187, 202, 216, and 217 (*p* = 0.529); 124, 125, 166, 179, 185, 200, and 203 (*p* = 0.519) and 103, 143, 159, 190, 191, 65, 152, and 201 (*p* = 0.509); 65, 152 and 201 (*p* = 0.5). The primary selection criterion was a probability of activity above 0.50 in the developed classification model, and the second criterion considered the availability of the substance and ease of acquisition. Consequently, of the 86 compounds selected via the model, it was only feasible to obtain a sufficient quantity for the in vitro testing of the compounds (60) betulinic acid (*p* = 0.560) and (136) lupeol (*p* = 0.560). To broaden the test series for the experimental validation of the model, two synthetic derivatives of the selected compounds were introduced. This aimed to evaluate whether modifications made through the insertion of acetate and methyl ester groups contributed to greater activity and enhanced cell viability. Therefore, the compounds (219) betulinic acid acetate and (220) betulinic acid methyl ester were included in the test series (Figure 1). We ran both compounds through our QSAR model to ensure they were predicted to be active before subjecting them to the in vitro assay.

According to our QSAR model, betulinic acid acetate (219) presented activity probability values of 0.57, while the compound betulinic acid methyl ester (220) exhibited probability values corresponding to 0.57. Hence, as these compounds showed activity probability values above 0.5 (random), they were subjected to in vitro testing, bringing the total to four compounds in the experimental validation of the study, namely the following: (60) betulinic acid (*p* = 0.560), (136) lupeol (*p* = 0.560), (219) betulinic acid acetate (*p* = 0.57) and (220) betulinic acid methyl ester (*p* = 0.57).

### 3.4. In Vitro Activity Assessment

A High-Content Screening (HCS) Assay was devised to evaluate compounds inhibiting infection and cytotoxicity in Vero cells infected with a SARS-CoV-2 isolate [13]. The potential antiviral activity of four terpene-class compounds against SARS-CoV-2 in Vero CCL-81 cells was evaluated. An initial screening was conducted and the compounds were tested at a single concentration of 10 µM, as indicated in Table 2.

Our results show that betulinic acid and its derivatives exhibited the highest percentages of inhibition. However, the derivatives betulinic acid methyl ester and betulinic acid acetate did not show high rates of cell viability. In contrast, betulinic acid demonstrated an inhibition of SARS-CoV-2 above 50% and maintained cell viability percentages above 70%. Lupeol showed inhibition percentages of 47.29%, indicating moderate activity, and a cell viability rate of 149.31% [13]. These findings demonstrate that the compounds under study showed the inhibitory potential of SARS-CoV-2 inhibition, thus being promising compounds in future studies.

### 3.5. Molecular Docking

Molecular docking simulations were conducted on our top compounds to further substantiate our predictions for these compounds to act as potential inhibitors of SARS-CoV-2. The molecular docking simulation aimed to show a proposed binding pose and estimate a binding affinity of the compounds (60) betulinic acid and (136) lupeol to targets related to this effect, including main protease (M-pro) enzymes (PDB: 5RG1), papain-like protease (PL-pro) (PDB: 7TZJ), spike protein (PDB: 8C8P) and RNA-dependent RNA polymerase (PDB: 6M71). Prior to the molecular docking simulation, a redocking procedure was carried out between the ligands and the co-crystallized proteins to validate our docking procedures (Figure S2). The redocking, including the RMSD (Root Mean Square Deviation) values which measure the deviation between the experimentally determined crystallographic structure and the coupled pose, are shown in Table S3 and Figure S3—Supplementary Materials [45,46]. Table 3 presents the affinity results for the compounds under study, according to the energetic values obtained from the Moldock Score algorithm (KJ·mol^−1^).

**Figure 2:**
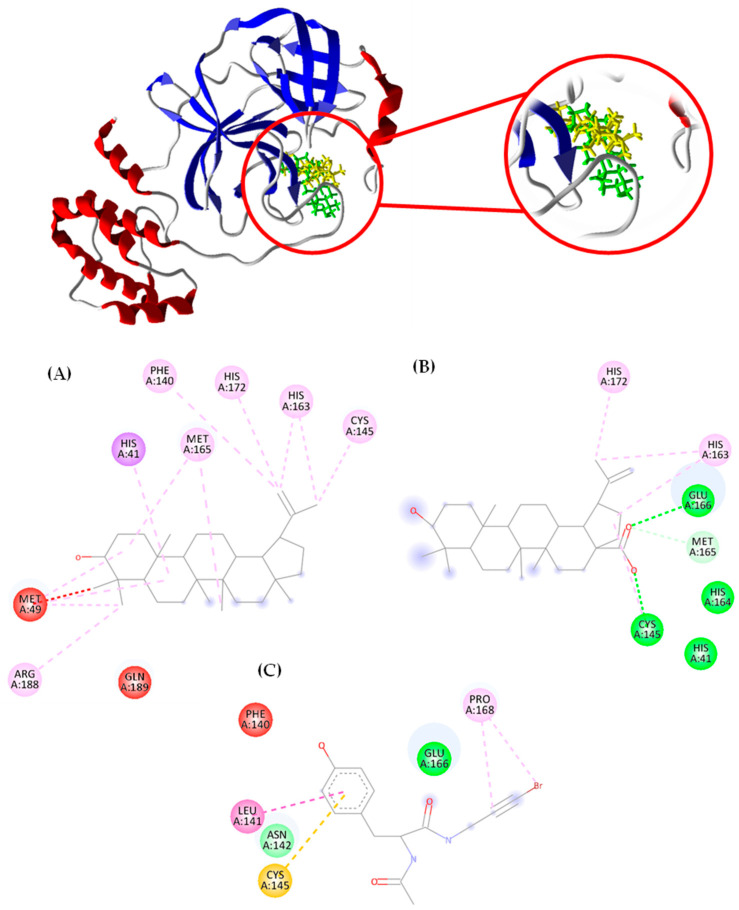
Two-dimensional molecular interactions established by the compounds lupeol (**A**), betulinic acid (**B**), and PDB ligand (**C**) with the target main protease (M-pro) (PDB: 5RG1). Residues: His (Histidine), Phe (Phenylalanine), Cys (Cysteine), Met (Methionine), Arg (Arginine), Gln (Glutamine), Glu (Glutamic Acid), Asn (Asparagine), Pro (Proline) and Leu (Leucine). Interactions: red (unfavorable bump), purple (pi-sigma), pink (alkyl, pi-alkyl, amide-pi stacked), dark green (conventional hydrogen bond), light green (carbon–hydrogen bond), orange (pi-anion and pi-sulfur) and blue (halogen—Cl, Br, I).

The compounds under investigation exhibit negative binding energy values for all targets, indicating their favorable interaction with the proteins. While the terpenes under study did not show a higher affinity than the PDB ligand in any of the enzymes, it is noteworthy that for the main protease (M-pro) (PDB: 5RG1) target, the compounds demonstrated binding energy values close to those of the controls, with affinity probability values exceeding 0.8. Specifically, lupeol exhibited −88.88 KJ·mol^−1^ (*p* = 0.84), betulinic acid showed −91.66 KJ·mol^−1^ (*p* = 0.87), the PDB ligand presented −103.03 KJ·mol^−1^ (*p* = 0.98), being the most stable compound, displayed −104.71 KJ·mol^−1^ (*p* = 1). Interestingly, betulinic acid demonstrated a favorable binding free energy and high probability of binding to papain-like protease (PDB: 7TZJ) at −97.57 KJ·mol^−1^ (*p* = 0.79). The M-pro enzyme was the target with the highest affinity demonstrated by the compounds under study. The description of the affinity of the compounds with the other targets is described in Section S1 of the Supplementary Materials. Figure 2 illustrates the interaction of the compounds: lupeol (A), betulinic acid (B), the control drug, and the PDB ligand (C) with the target main protease (M-pro) (PDB: 5RG1).

In Figure 2, the molecular interactions with the main protease (M-pro) (PDB: 5RG1) involved hydrogen bonds (green dashed lines), hydrophobic interactions (pink, blue and orange dashed lines) and steric interactions (red dashed lines).

In the interaction of the lupeol compound, the involvement of crucial residues for maintaining the enzyme’s activity was observed. For instance, residues His 41 and Cys 145 play a key role in forming the enzyme’s catalytic dyad and ensuring the complete dimerization of the active site. His residue 163 is important for the formation of the side chain. Met residue 165 contributes to the formation of the central monomer of the protein, representing a large hydrophobic cavity. The residues Met 49 and Gln 189 suggest a degree of plasticity in the enzyme’s side chains [38].

Similar to lupeol, the triterpene betulinic acid displayed interactions with crucial residues for maintaining the activity of the main protease enzyme. These included residues Met 165, His 163, and Pro 168, related to the plasticity of the enzyme’s side chain. The residue Glu 166 is involved in the dimerization of the chain and the formation of the catalytic dyad, while His164 is crucial for the formation of the central monomer of the hydrophobic enzyme [38]. Additionally, betulinic acid shared hydrogen bond interactions with residue Glu 166 with the PDB ligand.

## 4. Discussion

The Fabaceae family, or Leguminosae, is the third-largest family of angiosperm plants [47,48]. This family represents 770 genera and around 19,500 species, distributed across several subfamilies [49,50]. It is the largest family of plants in Brazil, with approximately 2834 species found in different ecosystems [51]. The Fabaceae family exhibits a rich diversity of chemical compounds, with a particular emphasis on phenolic compounds and alkaloids [52].

Terpenes are another significant group associated with numerous biological activities, including antimicrobial and antiviral activity [53]. Compounds within the terpene class are promising natural compounds for the creation of new antiviral agents [54,55,56,57,58]. Natural terpenoids and their synthetic analogues are considered valuable sources for novel medicines for the treatment of various diseases, due to their diverse molecular structures, low toxicity, and the ability to impact several specific cellular targets, resulting in a wide range of biological activities [58,59,60,61,62,63].

In silico studies indicate that triterpenes such as lupeol and betulinic acid have potential anti-SARS-CoV-2 activity [64]. Lupeol, found in several plants, is known for its anti-inflammatory, antioxidant and anticancer properties, and is being investigated as an inhibitor of the main protease (Mpro) of the virus, essential for its replication [65,66,67]. Molecular modeling suggests that lupeol can bind to the active site of Mpro, inhibiting its activity by interacting with amino acids such as cysteine and histidine [68]. In summary, lupeol shows potential as an inhibitor of Mpro of SARS-CoV-2, but to date its efficacy has not yet been experimentally validated. Similarly to lupeol, it was observed that betulinic acid in molecular docking studies and molecular dynamics simulations showed an inhibition of the activity of the enzymes M-protease and papain-like protease with high stability. These enzymes belong to the family of cysteine proteases, a type of enzyme which cleaves proteins through the hydrolysis of peptide bonds as they have a cysteine residue in the active center that acts as a nucleophile in the protein cleavage process [69]. Like lupeol, the use of betulinic acid for its anti-SARS-CoV-2 activity requires further evidence; this affirms the importance of the present work, as it provides experimental evidence on the anti-SARS-CoV-2 activity and also addresses the machine learning methodology and studies focused on the ligand and structure approaches.

In this study, a classification model was developed to identify compounds with a probability of inhibiting SARS-CoV-2. The created model successfully identified 86 compounds that presented a probability of activity exceeding 50% (*p* = 0.5) and assessed their applicability domain. The selected compounds belong to classes of diterpenes, which have shown promise in treating or preventing viral infections caused by enveloped viruses that undergo hemagglutinin-mediated fusion with a host cell and/or the resulting symptoms. Previous research by Tret’ yakowa and collaborators (2022) [58] reported the synthesis of Mannich diterpenic bases as potential therapeutic agents for Influenza A and SARS-CoV-2. Another class identified was triterpenes, that are widely used in traditional herbal medicine, representing an interesting case of natural compounds that play an important role in plant defense. Triterpenes are known for their antiviral activity against various diseases, including human immunodeficiency virus 1 (HIV-1), hepatitis B virus (HBV), hepatitis C virus (HCV), influenza A virus (IAV), Ebola virus (EBOV) and SARS-CoV-2 [56,64,70,71,72,73]. The third class worth mentioning consists of rare monoterpenes substituted with osidic units, which have demonstrated the ability to bind and interfere with the functions of different proteins in the SARS-CoV-2 virus, including the main protease, endoribonuclease, ADP ribose phosphatase, RNA-dependent polymerase, and spike protein [74,75,76,77]. These compounds also impact human cell proteins crucial for viral internalization and replication, including angiotensin-converting enzymes and cellular proteases, transmembrane serine protease 2, cathepsin B, and cathepsin L [74,77,78,79,80].

We selected the compounds lupeol and betulinic acid based on their probability of activity and availability and subjected them to biological testing. Additionally, two synthetic derivatives of betulinic acid were included: betulinic acid acetate and betulinic acid methyl ester. This was in order to investigate whether the activity would be enhanced or reduced. The results revealed that betulinic acid exhibited the highest percentage of inhibition and demonstrated a high rate of cell survival. Lupeol showed moderate activity but exhibited good cell viability. However, the synthetic derivatives displayed a higher percentage of inhibition, but did not show favorable percentages of cell viability.

The main protease was identified as the target with the highest affinity, and the compounds exhibited greater stability concerning the evaluation of free energy parameters. Lupeol and betulinic acid, both terpenes, have substantial evidence in the literature supporting their anti-SARS-CoV-2 potential. Elkousy and collaborators (2022) [81] identified lupeol as a promising candidate for a therapeutic agent against SARS-CoV-2 through an in silico study involving the Castor Oil Plant (*Ricinus communis*). Betulinic acid is addressed by Patel and collaborators (2023) in an in silico study on the bioprospecting of *Rosmarinus officinalis* for M-protease [82].

## 5. Conclusions

The bibliographic study documented and standardized 217 compounds from the Fabaceae family with potential therapeutic activities for several emerging diseases. A predictive model was developed and successfully classified 83 compounds, including betulinum and lupeol, that demonstrated inhibitory activity against SARS-CoV-2, validating the model. The main protease enzyme emerged as the most likely target. From this perspective, it can be concluded that the virtual screening identified compounds with a high probability of activity and stability, corroborated using in vitro tests.

## Figures and Tables

**Table 1:** Summary of parameters corresponding to the results obtained for all models with VolSurf descriptors.

Specie	Validation	Specificity	Sensitivity	Accuracy	Precision	Recall	ROC	MCC
SARS-CoV-2	Test	0.75	0.759	0.75	0.75	0.75	0.855	0.5
	Cross	0.702	0.729	0.716	0.716	0.729	0.802	0.431

**Table 2:** Anti-SARS-CoV-2 activity using Vero CCL-81 cells high-content screening at 10 µM.

Compound	CS/%	AA/%
Lupeol	149.31	47.29
Betulinic acid	76.42	59.20
Betulinic acid methyl ester	30.99	77.41
Betulinic acid acetate	39.78	91.11

(CS: cell survival; AA: antiviral activity).

**Table 3:** Binding energy (KJ·mol^−1^) and affinity probability (*p*) values of the compounds under study with the SARS-CoV-2 enzymes ^1^.

Compounds	M-Pro (PDB: 5RG1)	PL-Pro (PDB: 7TZJ)	Spike (PDB: 8C8P)	RNA-Dependent RNA Polymerase (PDB: 6M71)
Lupeol	−88.88 (*p* = 0.86)	−80.46 (*p* = 0.65)	−96.91 (*p* = 0.64)	−81.97 (*p* = 0.46)
Betulinic acid	−91.66 (*p* = 0.88)	−97.57 (*p* = 0.79)	−102.35 (*p* = 0.68)	−79.97 (*p* = 0.45)
PDB Ligand/Positive control	−103.03 (*p* = 1)	** −123.07 ** ***p* = 1)**	** −149.45 ** ** (*p* = 1) **	** 176.59 ** ** (*p* = 1) **

^1^ The compound with the highest affinity is highlighted in bold.

## Data Availability

Data is contained within the article and Supplementary Materials.

## Supplementary Materials

The following supporting information can be downloaded at https://www.mdpi.com/article/10.3390/pharmaceutics16070912/s1: Table S1: Terpene derivatives found in the Fabaceae family; Table S2: Prediction of the anti-SARS-CoV-2 activity of terpenes isolated in the Fabaceae family according to VolSurf descriptors; Figure S1: Compounds that did not show reliability in the applicability domain in the model created with AlvaDesc descriptors; Figure S2: Compounds that presented activity probability values above 0.50 and reliable applicability domain in the prediction model created with AlvaDesc descriptors; *S.01. Molecular Docking*; Table S3: RMSD values for the protein selected in the study; Figure S3: Redocking of the co-crystallized ligands and their respective poses. (A) M-protease target (PDB: 5RG1) and papain-like protease target (PDB: 7TZJ).

## Nomenclature

Area Over the Curve analysis	ROC
Carbon Nuclear Resonance Spectrometry	C-NMR
*Coronilla varia*	CV
Dulbecco’s Modified Eagle’s Medium	DMEM
Human Coronaviruses	HCoVs
Hydrogen Nuclear Resonance Spectrometry	H-NMR
Infrared Spectrometry	IV
Main protease	Mpro or 3CLpro
Mass Spectrometry	MS
Matthews Correlation Coefficient	MCC
*Melilotus officinalis*	MO
Multiplicity of Infection	MOI
*Ononis spinosa*	OS
Operetta High-Content Analysis System equipment	HCS
Papain-like protease	PLpro
Phosphate-buffered saline	PBS
Protein Data Bank	PDB
Quantitative Structure–Activity Relationship	QSAR
Random Forest	RF
RNA-dependent RNA polymerase	RdRp
*Robinia pseudoacacia*	RP
Ultraviolet Spectrometry	UV
