# Supplementary material for: In Silico and In Vitro Studies of Terpenes from the Fabaceae Family Using the Phenotypic Screening Model against the SARS-CoV-2 Virus
Source: Pharmaceutics. 2024 Jul 9;16(7):912. doi: 10.3390/pharmaceutics16070912 (PMC11279753; doi:10.3390/pharmaceutics16070912)
Supplement: Supplementary file 1 [file pharmaceutics-16-00912-s001.zip › pharmaceutics-3068162-supplementary.pdf]

Supplementary Materials: In Silico and In Vitro Studies of Terpenes from the Fabaceae Family Using the Phenotypic Screening Model against the SARS-CoV-2 Virus

Natália Ferreira de Sousa, Gabrielly Diniz Duarte, Carolina Borsoi Moraes, Cecília Gomes Barbosa, Holli-Joi Martin, Nail N. Muratov, Yuri Mangueira do Nascimento, Luciana Scotti, Lúcio Holanda Gondim de Freitas-Júnior, José Maria Barbosa Filho and Marcus Tullius Scotti

Table S1. Terpene derivatives found in the Fabaceae family.

ID	Compound	SMILE	Class	Gender	Species	Reference
**01**	α-Trujene	CC(C)C12CC1C(C)=CC2	Monoterpene	Lathyrus	Lathyrus odoratus	[1]
**02**	(1R)-(1)-β-Pinene	CC1=CC[C@@H]2C[C@H]1C2(C)C	Monoterpene	Lathyrus	Lathyrus odoratus	[1]
**03**	(1S)-(1)-β-Pinene	CC1(C)[C@@H]2C[C@H]1C(=C)CC2	Monoterpene	Lathyrus	Lathyrus odoratus	[1]
**04**	Myrcene	CC(C)=CCCC(=C)C=C	Monoterpene	Lathyrus	Lathyrus odoratus	[1]
**05**	Ocimene	CC(C)=CCC=C(C)C=C	Monoterpene	Lathyrus	Lathyrus odoratus	[1]
**06**	α-Phellandrene	CC(C)C1CC=C(C)C=C1	Monoterpene	Lathyrus	Lathyrus odoratus	[1]
**07**	(+)-Limonene	CC(=C)[C@@H]1CCC(C)=CC1	Monoterpene	Lathyrus	Lathyrus odoratus	[1]
**08**	α-Terpinene	CC(C)C1=CC=C(C)CC1	Monoterpene	Lathyrus	Lathyrus odoratus	[1]
**09**	(+/-)-Limonene	CC(=C)[C@H]1CCC(C)=CC1	Monoterpene	Lathyrus	Lathyrus odoratus	[1]
**10**	β-Phellandrene	CC(C)C1CCC(=C)C=C1	Monoterpene	Lathyrus	Lathyrus odoratus	[1]
**11**	γ-Terpinene	CC(C)C1=CCC(C)=CC1	Monoterpene	Lathyrus	Lathyrus odoratus	[1]
**12**	Terpinolene	CC(C)=C1CCC(C)=CC1	Monoterpene	Lathyrus	Lathyrus odoratus	[1]
**13**	Linalol	CC(C)=CCCC(C)(O)C=C	Monoterpene	Lathyrus	Lathyrus odoratus	[1]
**14**	α-Terpineol	CC1=CCC(CC1)C(C)(C)O	Monoterpene	Lathyrus	Lathyrus odoratus	[1]
**15**	Geraniol	[H]\C(CO)=C(\C)CCC=C(C)C	Monoterpene	Lathyrus	Lathyrus odoratus	[1]
**16**	Nerol	[H]\C(CO)=C(/C)CCC=C(C)C	Monoterpene	Lathyrus	Lathyrus odoratus	[1]
**17**	α-Bergamotene	CC(C)=CCCC1(C)C2CC1C(C)=CC2	Sesquiterpene	Lathyrus	Lathyrus odoratus	[1]
**18**	(E)-β-Farnesene	CC(C)=CCCC(C)=CCCC(=C)C=C	Sesquiterpene	Lathyrus	Lathyrus odoratus	[1]
**19**	Himachalene	[H][C@]12C=C(C)CCC1=C(C)CCCC2(C)C	Sesquiterpene	Lathyrus	Lathyrus odoratus	[1]
**20**	Zingiberene	[H][C@@]1(CC=C(C)C=C1)[C@@H](C)CCC=C(C)C	Sesquiterpene	Lathyrus	Lathyrus odoratus	[1]
**21**	β-Bisabolene	CC(C)=CCCC(=C)[C@H]1CCC(C)=CC1	Sesquiterpene	Lathyrus	Lathyrus odoratus	[1]
**22**	α-Farnesene	[H]\C(C\C([H])=C(/C)C=C)=C(\C)CCC=C(C)C	Sesquiterpene	Lathyrus	Lathyrus odoratus	[1]
**23**	α-Curcumene	[H]C(C)(CCC=C(C)C)C1=CC=C(C)C=C1	Sesquiterpene	Lathyrus	Lathyrus odoratus	[1]
**24**	(+)-α-Longipinene	[H][C@@]12CC=C(C)[C@@]3([H])[C@@]1([H])C(C)(C)CCC[C@]23C	Sesquiterpene	Lathyrus	Lathyrus odoratus	[1]
**25**	(-)-α-Cedrene	[H]C12C[C@@]3(CC=C1C)[C@H](C)CC[C@@]3([H])C2(C)C	Sesquiterpene	Lathyrus	Lathyrus odoratus	[1]
**26**	β-Sesquiphellandrene	[H]C1(CCC(=C)C=C1)C(C)CCC=C(C)C	Sesquiterpene	Lathyrus	Lathyrus odoratus	[1]

**27**	α-Patchoulene	CC1CCC23C1CC(CC=C2C)C3(C)C	Sesquiterpene	Lathyrus	Lathyrus odoratus	[1]
**28**	Nerolidol	[H]\C(CCC(C)(O)C=C)=C(\C)CCC=C(C)C	Sesquiterpene	Lathyrus	Lathyrus odoratus	[1]
**29**	α-Bisabolol	[H]C1(CCC(C)=CC1)C(C)(O)CCC=C(C)C	Sesquiterpene	Lathyrus	Lathyrus odoratus	[1]
**30**	(-)-Alloaromadrene	[H][C@@]12CCC(=C)[C@@]3([H])CC[C@@H](C)[C@@]3([H])[C@]1([H])C2(C)C	Sesquiterpene	Lathyrus	Lathyrus odoratus	[1]
**31**	(+)-γ-Gurjunene	[H]C1(C)CCC2([H])C1=CC([H])(CCC2([H])C)C(C)=C	Sesquiterpene	Lathyrus	Lathyrus odoratus	[1]
**32**	(-)-α-Cubebene	[H]C12C(C)=CCC11C2([H])C([H])(CCC1([H])C)C(C)C	Sesquiterpene	Lathyrus	Lathyrus odoratus	[1]
**33**	(+)-Ylangene	CC(C)[C@@H]1CC[C@@]2(C)C3CC=C(C)C2C13	Sesquiterpene	Lathyrus	Lathyrus odoratus	[1]
**34**	(-)-α-Copaene	[H][C@]12C3CC=C(C)C1[C@@]3(C)CC[C@H]2C(C)C	Sesquiterpene	Lathyrus	Lathyrus odoratus	[1]
**35**	β-Elemene	CC(=C)[C@@H]1CC[C@@](C)(C=C)[C@@H](C1)C(C)=C	Sesquiterpene	Lathyrus	Lathyrus odoratus	[1]
**36**	(-)-α-Gurjunene	[H][C@@]12CC[C@@H](C)[C@@]3(C)CCC(C)=C3[C@]1([H])C2(C)C	Sesquiterpene	Lathyrus	Lathyrus odoratus	[1]
**37**	β-Caryophyllene	[H][C@]12CC(C)(C)[C@]1([H])CC\C(C)=C\CCC2=C	Sesquiterpene	Lathyrus	Lathyrus odoratus	[1]
**38**	(+)-Aromadendrene	[H][C@@]12CCC(=C)[C@]3([H])CC[C@@H](C)[C@@]3([H])[C@]1([H])C2(C)C	Sesquiterpene	Lathyrus	Lathyrus odoratus	[1]
**39**	β-Cadinene	[H][C@@]12CC(C)=CC[C@@]1([H])C(C)=CC[C@H]2C(C)C	Sesquiterpene	Lathyrus	Lathyrus odoratus	[1]
**40**	Cubebene	[H][C@]12[C@@H](CC[C@@H](C)[C@]11CCC(=C)[C@]21[H])C(C)C	Sesquiterpene	Lathyrus	Lathyrus odoratus	[1]
**41**	γ-Muurolene	[H][C@@]12CCC(C)=C[C@]1([H])[C@H](CCC2=C)C(C)C	Sesquiterpene	Lathyrus	Lathyrus odoratus	[1]
**42**	γ-Cadinene	[H][C@@]12CCC(C)=C[C@@]1([H])[C@@H](CCC2=C)C(C)C	Sesquiterpene	Lathyrus	Lathyrus odoratus	[1]
**43**	(+)-δ-Cadinene	[H][C@@]12C=C(C)CCC1=C(C)CC[C@H]2C(C)C	Sesquiterpene	Lathyrus	Lathyrus odoratus	[1]
**44**	Calamenene	CC(C)[C@@H]1CC[C@H](C)C2=C1C=C(C)C=C2	Sesquiterpene	Lathyrus	Lathyrus odoratus	[1]
**45**	α-Muurolene	[H][C@]12CCC(C)=C[C@@]1([H])[C@@H](CC=C2C)C(C)C	Sesquiterpene	Lathyrus	Lathyrus odoratus	[1]
**46**	α-Caryophyllene	C\C1=C\CC(C)(C)\C=C/C\C(C)=C/CC1	Sesquiterpene	Lathyrus	Lathyrus odoratus	[1]
**47**	α-Selinene	[H][C@@]12C[C@@H](CC[C@@]1(C)CCC=C2C)C(C)=C	Sesquiterpene	Lathyrus	Lathyrus odoratus	[1]
**48**	(+)-Sativene	[H]C1[C@@H](CC[C@]2(C)C(=C)[C@@]3([H])CCC123)C(C)C	Sesquiterpene	Lathyrus	Lathyrus odoratus	[1]
**49**	Longifolene	CC12CCCC(C)(C)C3C(CCC13)C2=C	Sesquiterpene	Lathyrus	Lathyrus odoratus	[1]
**50**	Humulene	[H]\C1=C(C)\CCCC(=C)C2CC(C)(C)C2C1	Sesquiterpene	Lathyrus	Lathyrus odoratus	[1]
**51**	Guaiene	[H]C1(C)CCC2=C1CC(CCC2([H])C)=C(C)C	Sesquiterpene	Lathyrus	Lathyrus odoratus	[1]
**52**	Lanceol	[H]\C(CCC(=C)C1CCC(C)=CC1)=C(\C)CO	Sesquiterpene	Lathyrus	Lathyrus odoratus	[1]

**53**	Citronelal	[H]C(=O)CC(C)CCC=C(C)C	Monoterpene	Glycyrrhyza	Glycyrrhiza glabra var. glandulifera Rgl. Et Herd.	[2]
**54**	Carvone	CC(=C)[C@H]1CC=C(C)C(=O)C1	Monoterpene	Glycyrrhyza	Glycyrrhiza glabra var. glandulifera Rgl. Et Herd.	[2]
**55**	Bornyl Acetate	CC(=O)O[C@@H]1C[C@@H]2CC[C@@]1(C)C2(C)C	Sesquiterpene	Glycyrrhyza	Glycyrrhiza glabra var. glandulifera Rgl. Et Herd.	[2]
**56**	trans-Dihydrocarvone	C[C@H]1CC[C@@H](CC1=O)C(C)=C	Monoterpene	Glycyrrhyza	Glycyrrhiza glabra var. glandulifera Rgl. Et Herd.	[2]
**57**	Cis-Dihidrocarvone	C[C@@H]1CC[C@@H](CC1=O)C(C)=C	Monoterpene	Glycyrrhyza	Glycyrrhiza glabra var. glandulifera Rgl. Et Herd.	[2]
**58**	Endo-Borneol	[H]C1(O)CC2CCC1(C)C2(C)C	Sesquiterpene	Glycyrrhyza	Glycyrrhiza glabra var. glandulifera Rgl. Et Herd.	[2]
**59**	Isoborneol	CC1(C)[C@@H]2CC[C@@]1(C)[C@H](O)C2	Sesquiterpene	Glycyrrhyza	Glycyrrhiza glabra var. glandulifera Rgl. Et Herd.	[2]
**60**	Betulínic acid	[H][C@]12[C@@H](CC[C@@]1(CC[C@]1(C)[C@]2([H])CC[C@]2([H])[C@@]3(C)CC[C@H](O)C(C)(C)[C@]3([H])CC[C@@]12C)C(O)=O)C(C)=C	Triterpene	Acosmium	Acosmium bijugum	[3]
**61**	γ- elemene	CC(C)=C1CC[C@@](C)(C=C)[C@@H](C1)C(C)=C	Sesquiterpene	Pterodon	Pterodon emarginatus Vogel.	[4]
**62**	α-Humulene	[H]\C1=C([H])/C(C)(C)C\C([H])=C(C)\CC\C([H])=C(C)\C1	Sesquiterpene	Pterodon	Pterodon emarginatus Vogel.	[4]
**63**	Trans-alpha-bisabolol	[H][C@]1(CCC(C)=CC1)[C@](C)(O)CCC=C(C)C	Sesquiterpene	Pterodon	Pterodon emarginatus Vogel.	[4]

**64**	Allo-aromadendrene	[H]C12CCC(=C)[C@@]3([H])CC[C@@H](C)[C@@]3([H])C1([H])C2(C)C	Sesquiterpene	Pterodon	Pterodon emarginatus Vogel.	[4]
**65**	Cis-Sesquisabinene	[H]C(C)(CCC=C(C)C)C12CC1([H])[C@@](C)(CC2)OC	Sesquiterpene	Pterodon	Pterodon emarginatus Vogel.	[4]
**66**	Camphor	CC1(C)C2CCC1(C)C(=O)C2	Sesquiterpene	Ononis	Ononis natrix L.	[5]
**67**	Menthone	CC(C)[C@@H]1CC[C@@H](C)CC1=O	Monoterpene	Ononis	Ononis natrix L.	[5]
**68**	Terpinen-4-ol	CC(C)[C@]1(O)CCC(C)=CC1	Monoterpene	Ononis	Ononis natrix L.	[5]
**69**	Carvotanacetone	CC(C)[C@H]1CC=C(C)C(=O)C1	Monoterpene	Ononis	Ononis natrix L.	[5]
**70**	Thymol	CC(C)C1=C(O)C=C(C)C=C1	Monoterpene	Ononis	Ononis natrix L.	[5]
**71**	(Z)-Beta-Farnesene	[H]\C(CCC(=C)C=C)=C(/C)CCC=C(C)C	Sesquiterpene	Ononis	Ononis natrix L.	[5]
**72**	β-selinene	[H][C@@]12C[C@@H](CC[C@@]1(C)CCCC2=C)C(C)=C	Sesquiterpene	Ononis	Ononis natrix L.	[5]
**73**	Biclogermacrene	[H]\C1=C(C)/CC[C@]2([H])[C@]([H])(C([H])=C(C)CC1)C2(C)C	Sesquiterpene	Ononis	Ononis natrix L.	[5]
**74**	α-Muurolene	[H][C@@]12C=C(C)CC[C@]1(C)C(C)=CC[C@H]2C(C)C	Sesquiterpene	Ononis	Ononis natrix L.	[5]
**75**	Caryophyllene oxyde	[H][C@@]12CCC(=C)[C@@]3([H])CC(C)(C)[C@]3([H])CC[C@@]1(C)O2	Sesquiterpene	Ononis	Ononis natrix L.	[5]
**76**	10-epi-γ-eudesmol	[H][C@]1(CC[C@]2(C)CCCC(C)=C2C1)C(C)(C)O	Sesquiterpene	Ononis	Ononis natrix L.	[5]
**77**	γ-eudesmol	[H][C@]1(CC[C@@]2(C)CCCC(C)=C2C1)C(C)(C)O	Sesquiterpene	Ononis	Ononis natrix L.	[5]
**78**	β-eudesmol	[H][C@]1(CC[C@@]2(C)CCCC(=C)[C@]2([H])C1)C(C)(C)O	Sesquiterpene	Ononis	Ononis natrix L.	[5]
**79**	Delta-elemene	CC(C)C1=C[C@H](C(C)=C)[C@](C)(CC1)C=C	Sesquiterpene	Hymenaea	Hymenaea courbaril	[6]
**80**	β-humuleno	C\C1=C/CC(C)(C)\C=C\CC(=C)CCC1	Sesquiterpene	Hymenaea	Hymenaea courbaril	[6]
**81**	Germacrene D	[H]\C1=C(C)/CCC(C(C)C)\C([H])=C([H])\C(=C)CC1	Sesquiterpene	Hymenaea	Hymenaea courbaril	[6]
**82**	Germacrene B	[H]\C1=C(C)/CCC(CC([H])=C(C)CC1)=C(C)C	Sesquiterpene	Hymenaea	Hymenaea courbaril	[6]
**83**	Biciclo elemene	CC(=C)C1C2CC2CCC1(C)C=C	Sesquiterpene	Caesalpinia	Caesalpinia pulcherrima	[6]
**84**	γ-cariophylene	[H]\C1=C(C)\CC[C@]2([H])[C@]([H])(CC2(C)C)C(=C)CC1	Sesquiterpene	Caesalpinia	Caesalpinia pulcherrima	[6]
**85**	β-copaene	[H][C@]12C3C(CCC1=C)[C@@]2(C)CC[C@H]3C(C)C	Sesquiterpene	Caesalpinia	Caesalpinia pulcherrima	[6]
**86**	Epsilon muurolene	[H]C1(CCC(=C)C2([H])CCC(=C)CC12[H])C(C)C	Sesquiterpene	Caesalpinia	Caesalpinia pulcherrima	[6]

**87**	Biciclogermacrene	[H]\C1=C(C)/CC[C@]2([H])[C@]([H])(\C([H])=C(C)\CC1)C2(C)C	Sesquiterpene	Caesalpinia	Caesalpinia pulcherrima	[6]
**88**	α-amorphene	[H][C@@]12CCC(C)=C[C@]1([H])[C@@H](CC=C2C)C(C)C	Sesquiterpene	Caesalpinia	Caesalpinia pulcherrima	[6]
**89**	Camphene	CC1(C)[C@@H]2CC[C@@H](C2)C1=C	Monoterpene	Cordealxia	Cordealxia edulis	[7]
**90**	Verbenene	[H]C12C(=C)C=CC11CC1C2(C)C	Monoterpene	Cordealxia	Cordealxia edulis	[7]
**91**	Sabinene	CC(C)C12CC1C(=C)CC2	Monoterpene	Cordealxia	Cordealxia edulis	[7]
**92**	β-pinene	[H]C12CC11CCC(=C)C1([H])C2(C)C	Monoterpene	Cordealxia	Cordealxia edulis	[7]
**93**	Delta-3-carene	CC1=CCC2C(C1)C2(C)C	Monoterpene	Cordealxia	Cordealxia edulis	[7]
**94**	1,8 Cineole	C[C@@]12CCC(CC1)C(C)(C)O2	Monoterpene	Cordealxia	Cordealxia edulis	[7]
**95**	(E) beta-ocimene	[H]\C(CC=C(C)C)=C(\C)C=C	Monoterpene	Cordealxia	Cordealxia edulis	[7]
**96**	Fenchyl-alcohol	C[C@@H]1C(C)(C)[C@@H]2CC[C@@]1(C)C2	Monoterpene	Cordealxia	Cordealxia edulis	[7]
**97**	Pinocarveol	[H]C12C(=C)C(O)CC11CC1C2(C)C	Monoterpene	Cordealxia	Cordealxia edulis	[7]
**98**	Pinocarvone	[H]C12C(=C)C(=O)CC11CC1C2(C)C	Monoterpene	Cordealxia	Cordealxia edulis	[7]
**99**	Myrtenol	CC1(C)C2CC1C(CO)=CC2	Monoterpene	Cordealxia	Cordealxia edulis	[7]
**100**	Myrtenal	[H]C(=O)C1=CCC23CC2C(C)(C)C13[H]	Monoterpene	Cordealxia	Cordealxia edulis	[7]
**101**	Ascaridole	CC(C)C12CCC(C)(OO1)C=C2	Monoterpene	Cordealxia	Cordealxia edulis	[7]
**102**	(E) Citral	[H]C(=O)C(\[H])=C(/C)CCC=C(C)C	Monoterpene	Cordealxia	Cordealxia edulis	[7]
**103**	Citronellyl acetate	CC(CCOC(C)=O)CCC=C(C)C	Monoterpene	Cordealxia	Cordealxia edulis	[7]
**104**	Geranyl acetate	[H]\C(COC(C)=O)=C(\C)CCC=C(C)C	Monoterpene	Cordealxia	Cordealxia edulis	[7]
**105**	Isoledene	CC1CCC2=C1C1C(CCC2C)C1(C)C	Sesquiterpene	Cordealxia	Cordealxia edulis	[7]
**106**	Alpha-ylangene	CC(C)C1CCC2(C)C3CC=C(C)C2C13	Sesquiterpene	Cordealxia	Cordealxia edulis	[7]
**107**	β-bourbonene	[H][C@@]12CCC(=C)[C@]1([H])[C@@]1([H])[C@@H](CC[C@@]21C)C(C)C	Sesquiterpene	Cordealxia	Cordealxia edulis	[7]
**108**	(E)-alpha-bergamotene	CC(C)=CCCC1(C)[C@@H]2C[C@H]1C(C)=CC2	Sesquiterpene	Cordealxia	Cordealxia edulis	[7]
**109**	Callarene	CC1CCC=C2CCC3C(C3(C)C)C12C	Sesquiterpene	Cordealxia	Cordealxia edulis	[7]
**110**	Ledene	[H][C@@]12CCC(C)=C3CC[C@@H](C)[C@@]3([H])[C@]1([H])C2(C)C	Sesquiterpene	Cordealxia	Cordealxia edulis	[7]
**111**	Eremophylenne	C[C@H]1CCC=C2CC[C@H](C[C@]12C)C(C)=C	Sesquiterpene	Cordealxia	Cordealxia edulis	[7]
**112**	Alpha-calacorene	CC(C)[C@@H]1CC=C(C)C2=C1C=C(C)C=C2	Sesquiterpene	Cordealxia	Cordealxia edulis	[7]
**113**	(Z) Calamene	CC(C)[C@H]1CC[C@@H](C)C2=C1C=C(C)C=C2	Sesquiterpene	Cordealxia	Cordealxia edulis	[7]

**114**	Cadina-1,4-diene	CC(C)C1CC[C@H](C)C2=CCC(C)=CC12	Sesquiterpene	Cordealxia	Cordealxia edulis	[7]
**115**	Spathulenol	[H][C@@]12CCC(=C)[C@]3([H])CC[C@](C)(O)[C@@]3([H])[C@]1([H])C2(C)C	Sesquiterpene	Cordealxia	Cordealxia edulis	[7]
**116**	Palustrol	CC1CCC2(O)C1C1C(CCC2C)C1(C)C	Sesquiterpene	Cordealxia	Cordealxia edulis	[7]
**117**	Vidiflorol	[H][C@@]12CC[C@](C)(O)[C@@]3([H])CC[C@@H](C)[C@@]3([H])[C@]1([H])C2(C)C	Sesquiterpene	Cordealxia	Cordealxia edulis	[7]
**118**	Ledol	[H][C@@]12CC[C@@](C)(O)[C@@]3([H])CC[C@@H](C)[C@@]3([H])[C@]1([H])C2(C)C	Sesquiterpene	Cordealxia	Cordealxia edulis	[7]
**119**	Diterpen CM1 13	CC(=O)O[C@@H]1CCC2[C@H](CC\C(C)=C\C(O)=O)C(=C)CCC2C1(C)C	Diterpene	Copaifera	Copaifera Multiguja Haine	[8]
**120**	Diterpene CM2 14	C\C(CC[C@H]1C2CC[C@@H](O)C(C)(C)C2CCC1=C)=C/C(O)=O	Diterpene	Copaifera	Copaifera Multiguja Haine	[8]
**121**	Diterpene CM4 15	[H]C1CCC2[C@H](CC\C(C)=C\C(O)=O)C(=C)CCC2C1(C)C	Diterpene	Copaifera	Copaifera Multiguja Haine	[8]
**122**	Diterpene CM7 16	[H]C1CCC2[C@H](CC\C(C)=C\C(O)=O)C(=C)CCC2[C@]1(C)C(O)=O	Diterpene	Copaifera	Copaifera Multiguja Haine	[8]
**123**	Diterpene CM8 17	C\C(CC[C@H]1C2CC[C@@H](O)[C@@](C)(CO)C2CCC1=C)=C/C(O)=O	Diterpene	Copaifera	Copaifera Multiguja Haine	[8]
**124**	Diterpene CP1 21	[H]C1C[C@@]2(C)C(CCC=C2C(O)=O)[C@](C)(CCC2=COC=C2)[C@H]1C	Diterpene	Copaifera	Copaifera Multiguja Haine	[8]
**125**	Diterpene CP3 22	C[C@H]1[C@H](C[C@@]2(C)C(CCC=C2C(O)=O)[C@]1(C)CCC1=COC=C1)OC(C)=O	Diterpene	Copaifera	Copaifera Multiguja Haine	[8]
**126**	Diterpene CP2 23	[H][C@]12CC=C(C)[C@@H](CC\C(C)=C\C(O)=O)[C@]1(C)CCCC2(C)C	Diterpene	Copaifera	Copaifera Multiguja Haine	[8]
**127**	Diterpene CP4 24	[H][C@@]12CCC=C(C(O)=O)[C@@]1(C)CC[C@H](C)[C@@]2(C)CCC(O)CC(O)=O	Diterpene	Copaifera	Copaifera Multiguja Haine	[8]
**128**	Diterpene CT11 25	[H][C@@]12CCC=C(C(O)=O)[C@@]1(C)CC[C@H](C)[C@@]2(C)CC\C(CO)=C\C(O)=O	Diterpene	Copaifera	Copaifera Multiguja Haine	[8]

**129**	Diterpene CT12 26	[H][C@@]12CCCC(C)(C)C1=CCC(C)[C@H]2CC\C(C)=C\C(O)=O	Diterpene	Copaifera	Copaifera Multiguja Haine	[8]
**130**	Caryophyllene alcohol	CC1(C)CC2C1CCC1(C)CCCC2(O)C1	Sesquiterpene	Hymenaea	Hymenaea stigonocarpa	[9]
**131**	Labdan dienol acetate	[H][C@@]12CC=C(C)[C@H](CC\C(C)=C\COC(C)=C)[C@@]1(C)CCCC2(C)C	Diterpene	Hymenaea	Hymenaea stigonocarpa	[9]
**132**	Kaurenoic acid	[H][C@]12CC[C@@]34C[C@@H](CCC3[C@]1(C)CCC[C@@]2(C)C(O)=O)C(=C)C4	Diterpene	Copaifera	Copaifera langsdorffii	[10]
**133**	Camphene Hydrate	CC1(C)C2CCC(C2)C1(C)O	Monoterpene	Rhynchosia	Rhynchosia heynei	[11]
**134**	Mentha-1,8-dien-4-ol	CC(=C)[C@@]1(O)CCC(C)=CC1	Monoterpene	Rhynchosia	Rhynchosia heynei	[11]
**135**	Homopodatiline	[H][C@]12[C@@H](CC[C@]1(C)CC[C@]1(C)C2CCC2[C@@]3(C)CC[C@H](O)C(C)(C)C3CC[C@@]12C)C(C)=C	Triterpene	Acosmium	Acosmiun panamense (benth)	[12]
**136**	Lupeol	[H][C@]12[C@@H](CC[C@]1(C)CC[C@]1(C)[C@]2([H])CC[C@]2([H])C3CC[C@H](O)C(C)(C)[C@]3([H])CC[C@@]12C)C(C)=C	Triterpene	Albizia	Albizia coriaria Welw ex Oliver	[13]
**137**	Lupenone	[H][C@]12[C@@H](CC[C@]1(C)CC[C@]1(C)[C@]2([H])CC[C@]2([H])[C@@]3(C)C=CC(=O)C(C)(C)[C@]3([H])CC[C@@]12C)C(C)C	Triterpene	Albizia	Albizia coriaria Welw ex Oliver	[13]
**138**	Acacic acid lactone	CC1C23CCC4C(C)(C)C(O)CCC4(C)C2CC=C2C4CC(C)(C)C(O)CC4(C(O)CC132)C(O)=O	Triterpene	Albizia	Albizia coriaria Welw ex Oliver	[13]
**139**	P-cymene	CC(C)C1=CC=C(C)C=C1	Monoterpene	Pterocarpus	Pterocarpus soyauxii TAUB	[14]
**140**	Artemisia Ketone	CC(C)=CC(=O)C(C)(C)C=C	Monoterpene	Pterocarpus	Pterocarpus soyauxii TAUB	[14]
**141**	Cis-linalool oxide	CC(O)(CCC1OC1(C)C)C=C	Monoterpene	Pterocarpus	Pterocarpus soyauxii TAUB	[14]
**142**	Beta citral cycle	[H]C(=O)C1=C(C)CCCC1(C)C	Monoterpene	Pterocarpus	Pterocarpus soyauxii TAUB	[14]
**143**	Lynalil acetate	CC(C)=CCC[C@@](C)(OC(C)=O)C=C	Monoterpene	Pterocarpus	Pterocarpus soyauxii TAUB	[14]

**144**	Carvacrol	CC(C)C1=CC(O)=C(C)C=C1	Monoterpene	Pterocarpus	Pterocarpus soyauxii TAUB	[14]
**145**	7-ephi-sequithujene	[H][C@@]12C[C@@]1(CC=C2C)[C@H](C)CCC=C(C)C	Sesquiterpene	Pterocarpus	Pterocarpus soyauxii TAUB	[14]
**146**	β-funebrene	C[C@@H]1CC[C@H]2C(C)(C)[C@@H]3C[C@]12CCC3=C	Sesquiterpene	Pterocarpus	Pterocarpus soyauxii TAUB	[14]
**147**	5,9-Undecadien-2-one	CC(C)=CCC\C(C)=C\CCC(C)=O	Sesquiterpene	Pterocarpus	Pterocarpus soyauxii TAUB	[14]
**148**	Cis-cadina-1,4-diene	[H][C@@]12CC[C@H](C)CC1=C(CC=C2C)C(C)C	Monoterpene	Pterocarpus	Pterocarpus soyauxii TAUB	[14]
**149**	Beta-acoradiene	C[C@H]1CC[C@H](C(C)=C)[C@@]11CCC(C)=CC1	Monoterpene	Pterocarpus	Pterocarpus soyauxii TAUB	[14]
**150**	(E)-beta-ionone	[H]\C(=C(\[H])C1=C(C)CCCC1(C)C)C(C)=O	Sesquiterpene	Pterocarpus	Pterocarpus soyauxii TAUB	[14]
**151**	Beta allaskene	C[C@H]1CCC(=C(C)C)[C@]11CCC(C)=CC1	Sesquiterpene	Pterocarpus	Pterocarpus soyauxii TAUB	[14]
**152**	γ-Bisabolene	CC(C)=CCC\C(C)=C1/CCC(C)=CC1	Sesquiterpene	Pterocarpus	Pterocarpus soyauxii TAUB	[14]
**153**	Beta-copaen-4-alpha-ol	CC(C)[C@]1(O)C2CC2(C)C2CCC(=C)CC12	Sesquiterpene	Pterocarpus	Pterocarpus soyauxii TAUB	[14]
**154**	Eusdesma-4(15),7-dien-1-b-ol	CC(C)C1=CC[C@@]2(C)[C@H](O)CCC(=C)C2C1	Sesquiterpene	Pterocarpus	Pterocarpus soyauxii TAUB	[14]
**155**	Hexahydrofarnesyl acetone	CC(C)CCCC(C)CCCC(C)CCCC(C)=O	Sesquiterpene	Pterocarpus	Pterocarpus soyauxii TAUB	[14]
**156**	Farnesyl acetone	[H]\C(CC\C([H])=C(/C)CCC=C(C)C)=C(\[H])CCC(C)=O	Sesquiterpene	Pterocarpus	Pterocarpus soyauxii TAUB	[14]
**157**	Phytol	[H]\C(CO)=C(\C)CCC[C@H](C)CCC[C@H](C)CCCC(C)C	Alchol diterpene	Pterocarpus	Pterocarpus soyauxii TAUB	[14]

**158**	Alpha-cadinol	[H][C@@]12C=C(C)CC[C@@]1([H])[C@](C)(O)CC[C@H]2C(C)C	Sesquiterpene	Pongamia	Pongamia pinnata (L.) Pierre ou Millettia pinnata	[15]
**159**	Caryophyllene oxide	C[C@@]12CCC[C@H]3[C@H](CC3(C)C)C(=C)C[C@H]1O2	Sesquiterpene	Pongamia	Pongamia pinnata (L.) Pierre ou Millettia pinnata	[15]
**160**	Cycloart-23-ene-3beta,25-diol	[H]\C(C[C@@H](C)[C@@]1([H])CC[C@@]2(C)[C@]3([H])CC[C@]4([H])[C@]5(C[C@@]35CC[C@]12C)CC[C@H](O)C4(C)C)=C(\[H])C(C)(C)O	Triterpene	Pongamia	Pongamia pinnata (L.) Pierre ou Millettia pinnata	[15]
**161**	Friedelin	[H][C@@]12CCC(=O)[C@H](C)[C@@]1(C)CC[C@@]1([H])[C@@]2(C)CC[C@@]2(C)[C@]3([H])CC(C)(C)CC[C@]3(C)CC[C@]12C	Triterpene	Pongamia	Pongamia pinnata (L.) Pierre ou Millettia pinnata	[15]
**162**	Cys Phytol	[H]\C(CO)=C(/C)CCC[C@H](C)CCC[C@H](C)CCCC(C)C	Sesquiterpene	Trifolium	Trifolium pratense L. ssp. Nivale (Sieber) Asch. Et Gr	[16]
**163**	Alpha linalool	CC(=C)CCCC(C)(O)C=C	Monoterpene	Caesalpinia	Caesalpinia coriaria	[17]
**164**	p-menth-1-em-ol	CC(C)C1CCC(C)=CC1O	Monoterpene	Caesalpinia	Caesalpinia coriaria	[17]
**165**	Citronelol	C[C@@H](CCO)CCC=C(C)C	Monoterpene	Caesalpinia	Caesalpinia coriaria	[17]
**166**	Cassane 1	[H][C@@]12CCC(C=C)=C(C)[C@@]1([H])O[C@H](O)C[C@]1([H])C(C)(C)CCC[C@@]21C	Diterpene	Acacia	Acacia jaquemontii	[18]
**167**	Cassane 2	[H]C(=O)C1=C(CC[C@]2([H])[C@]1([H])O[C@H](O)C[C@]1([H])C(C)(C)CCC[C@@]21C)C=C	Diterpene	Acacia	Acacia jaquemontii	[18]
**168**	(13E)-labd-13-en-3Beta, 8alpha, 15-triol (24)	[H][C@]1(CC\C(C)=C\CO)[C@](C)(O)CC[C@@]2([H])C(C)(C)[C@@H](O)CC[C@]12C	Diterpene	Acacia	Acacia jaquemontii	[18]
**169**	(13E)-3B,8a-dihydroxylabd-13-em-15-oic acid (25)	[H][C@]1(CC\C(C)=C\C(O)=O)[C@](C)(O)CC[C@@]2([H])C(C)(C)[C@@H](O)CC[C@]12C	Diterpene	Acacia	Acacia jaquemontii	[18]
**170**	Acacid acid	CC1(C)CC2C(C[C@@H]1O)[C@](O)(C[C@]1(C)C2=CCC2[C@@]3(C)CC[C@H](O)C(C)(C)C3CC[C@@]12C)C(O)=O	Triterpene	Acacia sinuata	Acassia rossei	[18]

**171**	Sapogenin B (27)	CC1(C)CC2C3=CCC4[C@@]5(C)CC[C@H](O)C(C)(C)C5CC[C@@]4(C)[C@]3(C)C[C@@H](O)[C@@]22C[C@@H]1CC2=O	Triterpene	Acacia sinuata	Acassia rossei	[18]
**172**	Acacidiol (28)	CC1(C)CC2C(C[C@@H]1O)=CC[C@]1(C)C2=CCC2[C@@]3(C)CC[C@H](O)C(C)(C)C3CC[C@@]12C	Triterpene	Acacia sinuata	Acassia rossei	[18]
**173**	Acacigen B (29)	[H]\C(C1C\C(CO1)=C\C)=C(\C)C(=O)O[C@H]1C[C@@]2([C@H](O)C[C@]3(C)C(CCC4[C@@]5(C)CC[C@H](O)C(C)(C)C5CC[C@@]34C)C2CC1(C)C)C(O)=O	Triterpene	Acacia sinuata	Acassia rossei	[18]
**174**	(Z) Jasmone	[H]\C(CC)=C(/[H])CC1=C(C)CCC1=O	Sesquiterpene	Glycine	Glycine max	[19]
**175**	(E)-4,8-dimethyl-1,3,7-nonatriene	[H]\C(C=C)=C(\C)CCC=C(C)C	Monoterpene	Glycine	Glycine max	[19]
**176**	(Z)-4,8-dimethyl-1,3,7-nonatriene	[H]\C(C=C)=C(/C)CCC=C(C)C	Monoterpene	Glycine	Glycine max	[19]
**177**	Germacrene A	[H]\C1=C(C)/CC[C@@H](C\C([H])=C(C)\CC1)C(C)=C	Sesquiterpene	Glycine	Glycine max	[19]
**178**	Dihydroagathic acid	CC(CCC1C(=C)CCC2C1(C)CCCC2(C)C(O)=O)CC(O)=O	Diterpene	Copaifera	Copaifera duckei Dwyer	[20]
**179**	Agathic acid	[H]\C(=C(\C)CC[C@H]1C(=C)CC[C@]2([H])[C@]1(C)CCC[C@]2(C)C(O)=O)C(O)=O	Diterpene	Copaifera	Copaifera duckei Dwyer	[20]
**180**	Ent-agathic acid methyl ester	[H]\C(=C(\C)CC[C@@H]1C(=C)CC[C@]2([H])C(CCC[C@@]12C)C(=O)OC)C(=O)OC	Diterpene	Copaifera	Copaifera duckei Dwyer	[20]
**181**	Alepterolic acid	[H]\C(=C(\C)CC[C@H]1C(=C)CC[C@@]2([H])C(C)(C)[C@@H](O)CC[C@]12C)C(O)=O	Diterpene	Copaifera	Copaifera duckei Dwyer	[20]
**182**	3 beta-hydroxycopalic acid	[H]\C(=C(\C)CC[C@@H]1C(=C)CC[C@]2([H])C(C)(C)[C@@H](O)CC[C@@]12C)C(O)=O	Diterpene	Copaifera	Copaifera duckei Dwyer	[20]
**183**	Polyalthic acid	[H][C@]12CCC(=C)[C@@H](CCC3=COC=C3)[C@]1(C)CCC[C@]2(C)C(O)=O	Diterpene	Copaifera	Copaifera duckei Dwyer	[20]
**184**	Lambertianic acid	C[C@]12CCC[C@@](C)(C1CCC(=C)[C@H]2CCC1=COC=C1)C(O)=O	Diterpene	Copaifera	Copaifera duckei Dwyer	[20]
**185**	Hardwickii acid	[H][C@@]12CCC=C(C(O)=O)[C@@]1(C)CC[C@H](C)[C@@]2(C)CCC1=COC=C1	Diterpene	Copaifera	Copaifera duckei Dwyer	[20]

**186**	Unreleased compound 01	CC1CC=CC2=C1CCC(C2)=C(C)C	Sesquiterpene	Christia	Christia vespertilionis	[21]
**187**	Unreleased compound 01	CCCCCCCCC(O)COC(=O)CCCCCC\C=C\C=C\C=C\CC	Sesquiterpene	Christia	Christia vespertilionis	[21]
**188**	Delta-2-carene	[H][C@@]12CCC(C)=C[C@]1([H])C2(C)C	Sesquiterpee	Medicago	Medicago truncatula	[22]
**189**	Carvone oxide	[H][C@]12C[C@H](CC(=O)[C@@]1(C)O2)C(C)=C	Sesquiterpee	Hymenaea	Hymenaea verrucosa	[23]
**190**	Cyclopside 1	C\C(=C/CCC(C)(O[C@H]1OC(CO)[C@@H](OC(C)(CC\C=C(\C)C(O)=O)C=C)[C@@H](O)C1O)C=C)C(O)=O	Unusual monoterpene acid glycoside	Acacia	Acacia Cyclops	[24]
**191**	Ciclopside 2	CC1O[C@H](OC(C)(CC\C=C(/C)C(O)=O)C=C)[C@H](O)C(O)[C@@H]1OC(C)(CC\C=C(/C)C(O)=O)C=C	Unusual monoterpene acid glycoside	Acacia	Acacia Cyclops	[24]
**192**	Compound 175 (S)	[H][C@]1(CC[C@@](C)(O)C=C)[C@](C)(O)CC[C@@]2([H])C(C)(C)CCC[C@]12C	Diterpen	Acacia	Acacia rossei	[24]
**193**	Composto 176 R	[H][C@]1(CC[C@](C)(O)C=C)[C@](C)(O)CC[C@@]2([H])C(C)(C)CCC[C@]12C	Diterpen	Acacia	Acacia rossei	[24]
**194**	Composto 177	[H][C@]1(CC\C(C)=C\CO)[C@](C)(O)CC[C@@]2([H])C(C)(C)CCC[C@]12C	Diterpen	Acacia	Acacia rossei	[24]
**195**	Oleanoic Acid	[H]C1C[C@]2(C)C(=CC[C@]3([H])[C@@]4(C)CC[C@H](O)C(C)(C)[C@]4([H])CC[C@@]23C)[C@]2([H])CC(C)(C)CC[C@]12C(O)=O	Triterpene	Acacia	Acacia pulchella	[24]
**196**	Ácido equinocístico	[H][C@@]12CC(C)(C)CC[C@@]1([C@H](O)C[C@]1(C)C2=CC[C@]2([H])[C@@]3(C)CC[C@H](O)C(C)(C)[C@]3([H])CC[C@@]12C)C(O)=O	Triterpene	Acacia	Acacia pulchella	[24]
**197**	3,21-dioxo-olean-18-en-oic acid 182	CC1(C)C=C2C3CCC4[C@@]5(C)CCC(=O)C(C)(C)C5CC[C@@]4(C)[C@]3(C)CC[C@]2(CC1=O)C(O)=O	Triterpene	Acacia	Acacia pulchella	[24]
**198**	Ácido corosolico 183	C[C@@H]1CC[C@@]2(CC[C@]3(C)C(=CCC4[C@@]5(C)C[C@@H](C)[C@H](O)C(C)(C)C5CC[C@@]34C)C2[C@H]1C)C(O)=O	Triterpene	Acacia	Acacia auriculiformis	[24]
**199**	Lupenil palmitato 186	[H][C@]12[C@@H](CC[C@]1(C)CC[C@]1(C)C2CC[C@]2([H])[C@@]3(C)CC[C@H](OC(=O)CCCCCCCCCCCCCCC)C(C)(C)[C@]3([H])OC[C@@]12C)C(C)=C	Triterpene	Acacia	Acacia dealbata	[24]

**200**	Lupenil cinamato 187	[H][C@]12[C@@H](CC[C@]1(C)CC[C@]1(C)C2CC[C@]2([H])[C@@]3(C)CC[C@H](OC(=O)\C=C\C4=CC=CC=C4)C(C)(C)[C@]3([H])OC[C@@]12C)C(C)=C	Triterpene	Acacia	Acacia dealbata	[24]
**201**	Alpha amyrin	[H][C@]12CC=C3[O@]4([H])CC(C)(C)CC[O@]4(C)CC[C@@]3(C)[C@]1(C)CCC1C(C)(C)[C@H](CC[C@]21C)OC(=O)\C=C/C1=CC=C(O)C=C1	Triterpene	Acacia	Acacia linarioides Benth	[24]
**202**	Beta- amyrin	[H][C@@]12CC(C)(C)CC[C@]1(C)CC[C@]1(C)C2=CC[C@]2([H])[C@@]3(C)CC[C@@H](OC(=O)\C=C\C4=CC=C(O)C=C4)C(C)(C)C3CC[C@@]12C	Triterpene	Acacia	Acacia linarioides Benth	[24]
**203**	Trans-hydroxycinnamoyl ester of lupeol	[H][C@]12CCC3C4[C@@H](CC[C@]4(C)CC[C@@]3(C)[C@]1(C)CCC1C(C)(C)[C@H](CC[C@]21C)OC(=O)\C=C\C1=CC=C(O)C=C1)C(C)=C	Triterpene	Acacia	Acacia trineura	[24]
**204**	erythrodiol	CC1(C)CC[C@]2(CO)CC[C@]3(C)C(=CCC4[C@@]5(C)CCC(O)C(C)(C)C5CC[C@@]34C)C2C1	Triterpene	Acacia	Acacia saligna	[24]
**205**	3-b-o- trans-p-coumaroyl-erythrodiol	CC1(C)CC[C@]2(CO)CC[C@]3(C)C(=CCC4[C@@]5(C)CCC(OC(=O)C=CC6=CC=C(O)C=C6)C(C)(C)C5CC[C@@]34C)C2C1	Triterpene	Acacia	Acacia saligna	[24]
**206**	Dihidroespinasterol	CCC(CCC(C)C1CCC2C3=CCC4CC(O)CCC4(C)C3CCC12C)C(C)C	Triterpene	Acacia	Acacia longifolia	[24]
**207**	Campestenone	CC(C)C(C)CCC(C)C1CCC2C3CC=C4CC(=O)CCC4(C)C3CCC12C	Triterpene	Mimosa	Mimosa artemisinina	[25]
**208**	19-O-trans-feruloyl-labd-8(17)	C[C@H](CCO)CC[C@@H]1C(=C)CCC2[C@](C)(COC(=O)\C=C\C3=CC=C(O)C(O)=C3)CCC[C@@]12C	Diterpene	Mimosa	Mimosa pudica	[25]
**209**	19-O-[(E)-30 ,40 -dimethoxy cinnamoyl]-labd-8(17)-en- 15,19-diol	COC1=CC=C(\C=C\C(=O)OC[C@]2(C)CCC[C@@]3(C)[C@H](CC[C@H](C)CCO)C(=C)CCC23)C=C1O	Diterpene	Mimosa	Mimosa pudica	[25]
**210**	Querataroic acid	[H][C@@]12C[C@@](C)(CO)CC[C@@]1(CC[C@]1(C)C2=CC[C@]2([H])[C@@]3(C)CC[C@H](O)C(C)(C)[C@]3([H])CC[C@@]12C)C(O)=O	Triterpene	Mimosa	Medicago sativa L.	[26]
**211**	Hederagenim	[H][C@@]12CC(C)(C)CC[C@@]1(CC[C@]1(C)C2=CC[C@]2([H])[C@@]3(C)CC[C@H](O)[C@@](C)(CO)[C@]3([H])CC[C@@]12C)C(O)=O	Triterpene	Mimosa	Medicago sativa L.	[26]
**212**	Bayogenin	[H][C@@]12CC(C)(C)CC[C@@]1(CC[C@]1(C)C2=CC[C@]2([H])[C@@]3(C)C[C@H](O)[C@H](O)[C@@](C)(CO)[C@]3([H])CC[C@@]12C)C(O)=O	Triterpene	Mimosa	Medicago sativa L.	[26]
**213**	Medicagenic acid	[H][C@@]12CC(C)(C)CC[C@@]1(CC[C@]1(C)C2=CC[C@]2([H])[C@@]3(C)C[C@H](O)[C@H](O)[C@@](C)(C(O)=O)[C@]3([H])CC[C@@]12C)C(O)=O	Triterpene	Mimosa	Medicago sativa L.	[26]

**214**	Zanhic acid	[H][C@@]12CC[C@]3(C)[C@]([H])(CC=C4C5CC(C)(C)CC[C@@]5([C@@H](O)C[C@@]34C)C(O)=O)[C@@]1(C)C[C@H](O)[C@H](O)[C@@]2(C)C(O)=O	Triterpene	Mimosa	Medicago sativa L.	[26]
**215**	Melilotiogenin metil ester	COC(=O)[C@@]1(C)CC2C3CCC4[C@@]5(C)CC[C@H](O)[C@](C)(CO)C5CC[C@@]4(C)[C@]3(C)CC[C@@]2(C)C(=O)C1	Triterpene	Mimosa	Medicago sativa L.	[26]
**216**	Kaur-16-ene	CC1(C)CCCC2(C)C3CCC4CC3(CC4=C)CCC12	Diterpene	Acacia	Acacia rigidula benth	[18]
**217**	Triterpenoid	[H][C@@]12CC(C)(C)CC[C@@]1(CC[C@]1(C)C2=CC[C@]2([H])[C@@]3(C)CC[C@H](O)[C@@](C)(COS(O)(=O)=O)[C@]3([H])CC[C@@]12C)C(O)=O	Triterpene	Mimosa	Mimosa pudica	[25]

Table S2. Prediction of the anti-SARS-CoV-2 activity of terpenes isolated in the Fabaceae Family according to AlvaDesc descriptors.

**ID**	**Compound**	**SMILE**	**Probability**	**Prediction**	**Applicability domain**
**01**	α-Trujene	CC(C)C12CC1C(C)=CC2	0.460000008	I	Unreliable
**02**	(1R)-(1)-β-Pinene	CC1=CC[C@@H]2C[C@H]1C2(C)C	0.479999989	I	Unreliable
**03**	(1S)-(1)-β-Pinene	CC1(C)[C@@H]2C[C@H]1C(=C)CC2	0.49000001	I	Unreliable
**04**	Myrcene	CC(C)=CCCC(=C)C=C	0.479999989	I	Unreliable
**05**	Ocimene	CC(C)=CCC=C(C)C=C	0.479999989	I	Unreliable
**06**	α-Phellandrene	CC(C)C1CC=C(C)C=C1	0.469999999	I	Unreliable
**07**	(+)-Limonene	CC(=C)[C@@H]1CCC(C)=CC1	0.5	A	Unreliable
**08**	α-Terpinene	CC(C)C1=CC=C(C)CC1	0.460000008	I	Unreliable
**09**	(+/-)-Limonene	CC(=C)[C@H]1CCC(C)=CC1	0.5	A	Unreliable
**10**	β-Phellandrene	CC(C)C1CCC(=C)C=C1	0.469999999	I	Unreliable
**11**	γ-Terpinene	CC(C)C1=CCC(C)=CC1	0.49000001	I	Unreliable
**12**	Terpinolene	CC(C)=C1CCC(C)=CC1	0.519999981	A	Unreliable
**13**	Linalol	CC(C)=CCCC(C)(O)C=C	0.579999983	A	Unreliable
**14**	α-Terpineol	CC1=CCC(CC1)C(C)(C)O	0.569999993	A	Unreliable
**15**	Geraniol	[H]\C(CO)=C(\C)CCC=C(C)C	0.579999983	A	Unreliable
**16**	Nerol	[H]\C(CO)=C(/C)CCC=C(C)C	0.579999983	A	Unreliable
**17**	α-Bergamotene	CC(C)=CCCC1(C)C2CC1C(C)=CC2	0.479999989	I	Unreliable

**18**	(E)-β-Farnesene	CC(C)=CCCC(C)=CCCC(=C)C=C	0.469999999	I	Reliable
**19**	Himachalene	[H][C@]12C=C(C)CCC1=C(C)CCCC2(C)C	0.5	A	Unreliable
**20**	Zingiberene	[H][C@@]1(CC=C(C)C=C1)[C@@H](C)CCC=C(C)C	0.460000008	I	Reliable
**21**	β-Bisabolene	CC(C)=CCCC(=C)[C@H]1CCC(C)=CC1	0.469999999	I	Reliable
**22**	α-Farnesene	[H]\C(C\C([H])=C(/C)C=C)=C(\C)CCC=C(C)C	0.49000001	I	Reliable
**23**	α-Curcumene	[H]C(C)(CCC=C(C)C)C1=CC=C(C)C=C1	0.479999989	I	Reliable
**24**	(+)-α-Longipinene	[H][C@@]12CC=C(C)[C@@]3([H])[C@@]1([H])C(C)(C)CCC[C@]23C	0.519999981	A	Unreliable
**25**	(-)-α-Cedrene	[H]C12C[C@@]3(CC=C1C)[C@H](C)CC[C@@]3([H])C2(C)C	0.5	A	Unreliable
**26**	β-Sesquiphellandrene	[H]C1(CCC(=C)C=C1)C(C)CCC=C(C)C	0.460000008	I	Reliable
**27**	α-Patchoulene	CC1CCC23C1CC(CC=C2C)C3(C)C	0.5	A	Unreliable
**28**	**Nerolidol**	**[H]\C(CCC(C)(O)C=C)=C(\C)CCC=C(C)C**	**0.560000002**	**A**	**Reliable**
**29**	**α-Bisabolol**	**[H]C1(CCC(C)=CC1)C(C)(O)CCC=C(C)C**	**0.540000021**	**A**	**Reliable**
**30**	(-)-Alloaromadrene	[H][C@@]12CCC(=C)[C@@]3([H])CC[C@@H](C)[C@@]3([H])[C@]1([H])C2(C)C	0.479999989	I	Unreliable
**31**	(+)-γ-Gurjunene	[H]C1(C)CCC2([H])C1=CC([H])(CCC2([H])C)C(C)=C	0.49000001	I	Unreliable
**32**	(-)-α-Cubebene	[H]C12C(C)=CCC11C2([H])C([H])(CCC1([H])C)C(C)C	0.479999989	I	Unreliable
**33**	(+)-Ylangene	CC(C)[C@@H]1CC[C@@]2(C)C3CC=C(C)C2C13	0.50999999	A	Unreliable
**34**	(-)-α-Copaene	[H][C@]12C3CC=C(C)C1[C@@]3(C)CC[C@H]2C(C)C	0.50999999	A	Unreliable
**35**	β-Elemene	CC(=C)[C@@H]1CC[C@@](C)(C=C)[C@@H](C1)C(C)=C	0.460000008	I	Reliable
**36**	(-)-α-Gurjunene	[H][C@@]12CC[C@@H](C)[C@@]3(C)CCC(C)=C3[C@]1([H])C2(C)C	0.50999999	A	Unreliable
**37**	β-Caryophyllene	[H][C@]12CC(C)(C)[C@]1([H])CC\C(C)=C\CCC2=C	0.479999989	I	Unreliable
**38**	(+)-Aromadendrene	[H][C@@]12CCC(=C)[C@]3([H])CC[C@@H](C)[C@@]3([H])[C@]1([H])C2(C)C	0.479999989	I	Unreliable
**39**	β-Cadinene	[H][C@@]12CC(C)=CC[C@@]1([H])C(C)=CC[C@H]2C(C)C	0.50999999	A	Unreliable
**40**	Cubebene	[H][C@]12[C@@H](CC[C@@H](C)[C@]11CCC(=C)[C@]21[H])C(C)C	0.469999999	I	Unreliable
**41**	γ-Muurolene	[H][C@@]12CCC(C)=C[C@]1([H])[C@H](CCC2=C)C(C)C	0.5	A	Unreliable
**42**	γ-Cadinene	[H][C@@]12CCC(C)=C[C@@]1([H])[C@@H](CCC2=C)C(C)C	0.5	A	Unreliable
**43**	(+)-δ-Cadinene	[H][C@@]12C=C(C)CCC1=C(C)CC[C@H]2C(C)C	0.50999999	A	Unreliable
**44**	Calamenene	CC(C)[C@@H]1CC[C@H](C)C2=C1C=C(C)C=C2	0.479999989	I	Unreliable
**45**	α-Muurolene	[H][C@]12CCC(C)=C[C@@]1([H])[C@@H](CC=C2C)C(C)C	0.50999999	A	Unreliable
**46**	α-Caryophyllene	C\C1=C\CC(C)(C)\C=C/C\C(C)=C/CC1	0.5	A	Unreliable

**47**	α-Selinene	[H][C@@]12C[C@@H](CC[C@@]1(C)CCC=C2C)C(C)=C	0.469999999	I	Unreliable
**48**	(+)-Sativene	[H]C1[C@@H](CC[C@]2(C)C(=C)[C@@]3([H])CCC123)C(C)C	0.5	A	Unreliable
**49**	Longifolene	CC12CCCC(C)(C)C3C(CCC13)C2=C	0.50999999	A	Unreliable
**50**	Humulene	[H]\C1=C(C)\CCCC(=C)C2CC(C)(C)C2C1	0.479999989	I	Unreliable
**51**	Guaiene	[H]C1(C)CCC2=C1CC(CCC2([H])C)=C(C)C	0.5	A	Unreliable
**52**	**Lanceol**	**[H]\C(CCC(=C)C1CCC(C)=CC1)=C(\C)CO**	**0.560000002**	**A**	**Reliable**
**53**	Citronelal	[H]C(=O)CC(C)CCC=C(C)C	0.529999971	A	Unreliable
**54**	Carvone	CC(=C)[C@H]1CC=C(C)C(=O)C1	0.560000002	A	Unreliable
**55**	Bornyl Acetate	CC(=O)O[C@@H]1C[C@@H]2CC[C@@]1(C)C2(C)C	0.519999981	A	Unreliable
**56**	trans-Dihydrocarvone	C[C@H]1CC[C@@H](CC1=O)C(C)=C	0.519999981	A	Unreliable
**57**	Cis-Dihidrocarvone	C[C@@H]1CC[C@@H](CC1=O)C(C)=C	0.519999981	A	Unreliable
**58**	Endo-Borneol	[H]C1(O)CC2CCC1(C)C2(C)C	0.560000002	A	Unreliable
**59**	Isoborneol	CC1(C)[C@@H]2CC[C@@]1(C)[C@H](O)C2	0.560000002	A	Unreliable
**60**	**Betulínic acid**	**[H][C@]12[C@@H](CC[C@@]1(CC[C@]1(C)[C@]2([H])CC[C@]2([H])[C@@]3(C)C C[C@H](O)C(C)(C)[C@]3([H])CC[C@@]12C)C(O)=O)C(C)=C**	**0.560000002**	**A**	**Reliable**
**61**	γ- elemene	CC(C)=C1CC[C@@](C)(C=C)[C@@H](C1)C(C)=C	0.469999999	I	Reliable
**62**	α-Humulene	[H]\C1=C([H])/C(C)(C)C\C([H])=C(C)\CC\C([H])=C(C)\C1	0.5	A	Unreliable
**63**	**Trans-alpha-bisabolol**	**[H][C@]1(CCC(C)=CC1)[C@](C)(O)CCC=C(C)C**	**0.540000021**	**A**	**Reliable**
**64**	Allo-aromadendrene	[H]C12CCC(=C)[C@@]3([H])CC[C@@H](C)[C@@]3([H])C1([H])C2(C)C	0.479999989	I	Unreliable
**65**	**Cis-Sesquisabinene**	**[H]C(C)(CCC=C(C)C)C12CC1([H])[C@@](C)(CC2)OC**	**0.5**	**A**	**Reliable**
**66**	Camphor	CC1(C)C2CCC1(C)C(=O)C2	0.519999981	A	Unreliable
**67**	Menthone	CC(C)[C@@H]1CC[C@@H](C)CC1=O	0.560000002	A	Unreliable
**68**	Terpinen-4-ol	CC(C)[C@]1(O)CCC(C)=CC1	0.569999993	A	Unreliable
**69**	Carvotanacetone	CC(C)[C@H]1CC=C(C)C(=O)C1	0.550000012	A	Unreliable
**70**	Thymol	CC(C)C1=C(O)C=C(C)C=C1	0.519999981	A	Unreliable
**71**	(Z)-Beta-Farnesene	[H]\C(CCC(=C)C=C)=C(/C)CCC=C(C)C	0.469999999	I	Reliable
**72**	β-selinene	[H][C@@]12C[C@@H](CC[C@@]1(C)CCCC2=C)C(C)=C	0.479999989	I	Unreliable
**73**	Biciclogermacrene	[H]\C1=C(C)/CC[C@]2([H])[C@]([H])(C([H])=C(C)CC1)C2(C)C	0.460000008	I	Unreliable
**74**	α-Muurolene	[H][C@@]12C=C(C)CC[C@]1(C)C(C)=CC[C@H]2C(C)C	0.519999981	A	Unreliable

**75**	Caryophyllene oxyde	[H][C@@]12CCC(=C)[C@@]3([H])CC(C)(C)[C@]3([H])CC[C@@]1(C)O2	0.50999999	A	Unreliable
**76**	**10-epi-γ-eudesmol**	**[H][C@]1(CC[C@]2(C)CCCC(C)=C2C1)C(C)(C)O**	**0.569999993**	**A**	**Reliable**
**77**	**γ-eudesmol**	**[H][C@]1(CC[C@@]2(C)CCCC(C)=C2C1)C(C)(C)O**	**0.569999993**	**A**	**Reliable**
**78**	**β-eudesmol**	**[H][C@]1(CC[C@@]2(C)CCCC(=C)[C@]2([H])C1)C(C)(C)O**	**0.560000002**	**A**	**Reliable**
**79**	Delta-elemene	CC(C)C1=C[C@H](C(C)=C)[C@](C)(CC1)C=C	0.479999989	I	Reliable
**80**	β-humuleno	C\C1=C/CC(C)(C)\C=C\CC(=C)CCC1	0.479999989	I	Unreliable
**81**	Germacrene D	[H]\C1=C(C)/CCC(C(C)C)\C([H])=C([H])\C(=C)CC1	0.460000008	I	Reliable
**82**	Germacrene B	[H]\C1=C(C)/CCC(CC([H])=C(C)CC1)=C(C)C	0.479999989	I	Unreliable
**83**	Biciclo elemene	CC(=C)C1C2CC2CCC1(C)C=C	0.460000008	I	Unreliable
**84**	γ-cariophylene	[H]\C1=C(C)\CC[C@]2([H])[C@]([H])(CC2(C)C)C(=C)CC1	0.479999989	I	Unreliable
**85**	β-copaene	[H][C@]12C3C(CCC1=C)[C@@]2(C)CC[C@H]3C(C)C	0.5	A	Unreliable
**86**	Epsilon muurolene	[H]C1(CCC(=C)C2([H])CCC(=C)CC12[H])C(C)C	0.50999999	A	Unreliable
**87**	Biciclogermacrene	[H]\C1=C(C)/CC[C@]2([H])[C@]([H])(\C([H])=C(C)\CC1)C2(C)C	0.460000008	I	Unreliable
**88**	α-amorphene	[H][C@@]12CCC(C)=C[C@]1([H])[C@@H](CC=C2C)C(C)C	0.50999999	A	Unreliable
**89**	Camphene	CC1(C)[C@@H]2CC[C@@H](C2)C1=C	0.479999989	I	Unreliable
**90**	Verbenene	[H]C12C(=C)C=CC11CC1C2(C)C	0.419999987	I	Unreliable
**91**	Sabinene	CC(C)C12CC1C(=C)CC2	0.460000008	I	Unreliable
**92**	β-pinene	[H]C12CC11CCC(=C)C1([H])C2(C)C	0.439999998	I	Unreliable
**93**	Delta-3-carene	CC1=CCC2C(C1)C2(C)C	0.460000008	I	Unreliable
**94**	1,8 Cineole	C[C@@]12CCC(CC1)C(C)(C)O2	0.479999989	I	Unreliable
**95**	(E) beta-ocimene	[H]\C(CC=C(C)C)=C(\C)C=C	0.479999989	I	Unreliable
**96**	Fenchyl-alcohol	C[C@@H]1C(C)(C)[C@@H]2CC[C@]1(C)C2	0.469999999	I	Unreliable
**97**	Pinocarveol	[H]C12C(=C)C(O)CC11CC1C2(C)C	0.519999981	A	Unreliable
**98**	Pinocarvone	[H]C12C(=C)C(=O)CC11CC1C2(C)C	0.540000021	A	Unreliable
**99**	Myrtenol	CC1(C)C2CC1C(CO)=CC2	0.560000002	A	Unreliable
**100**	Myrtenal	[H]C(=O)C1=CCC23CC2C(C)(C)C13[H]	0.49000001	I	Unreliable
**101**	Ascaridole	CC(C)C12CCC(C)(OO1)C=C2	0.540000021	A	Unreliable
**102**	(E) Citral	[H]C(=O)C(\[H])=C(/C)CCC=C(C)C	0.529999971	A	Unreliable
**103**	**Citronellyl acetate**	**CC(CCOC(C)=O)CCC=C(C)C**	**0.50999999**	**A**	**Reliable**

**104**	**Geranyl acetate**	**[H]\C(COC(C)=O)=C(\C)CCC=C(C)C**	**0.550000012**	**A**	**Reliable**
**105**	Isoledene	CC1CCC2=C1C1C(CCC2C)C1(C)C	0.479999989	I	Unreliable
**106**	Alpha-ylangene	CC(C)C1CCC2(C)C3CC=C(C)C2C13	0.50999999	A	Unreliable
**107**	β-bourbonene	[H][C@@]12CCC(=C)[C@]1([H])[C@@]1([H])[C@@H](CC[C@@]21C)C(C)C	0.49000001	I	Unreliable
**108**	(E)-alpha-bergamotene	CC(C)=CCCC1(C)[C@@H]2C[C@H]1C(C)=CC2	0.479999989	I	Unreliable
**109**	Callarene	CC1CCC=C2CCC3C(C3(C)C)C12C	0.479999989	I	Unreliable
**110**	Ledene	[H][C@@]12CCC(C)=C3CC[C@@H](C)[C@@]3([H])[C@]1([H])C2(C)C	0.49000001	I	Unreliable
**111**	Eremophylenne	C[C@H]1CCC=C2CC[C@H](C[C@]12C)C(C)=C	0.469999999	I	Unreliable
**112**	Alpha-calacorene	CC(C)[C@@H]1CC=C(C)C2=C1C=C(C)C=C2	0.460000008	I	Unreliable
**113**	(Z) Calamene	CC(C)[C@H]1CC[C@@H](C)C2=C1C=C(C)C=C2	0.479999989	I	Unreliable
**114**	Cadina-1,4-diene	CC(C)C1CC[C@H](C)C2=CCC(C)=CC12	0.50999999	A	Unreliable
**115**	Spathulenol	[H][C@@]12CCC(=C)[C@]3([H])CC[C@](C)(O)[C@@]3([H])[C@]1([H])C2(C)C	0.529999971	A	Unreliable
**116**	Palustrol	CC1CCC2(O)C1C1C(CCC2C)C1(C)C	0.529999971	A	Unreliable
**117**	Vidiflorol	[H][C@@]12CC[C@](C)(O)[C@@]3([H])CC[C@@H](C)[C@@]3([H])[C@]1([H])C2(C<br>)C	0.529999971	A	Unreliable
**118**	Ledol	[H][C@@]12CC[C@@](C)(O)[C@@]3([H])CC[C@@H](C)[C@@]3([H])[C@]1([H])C2(<br>C)C	0.529999971	A	Unreliable
**119**	**Diterpen CM1 13**	**CC(=O)O[C@@H]1CCC2[C@H](CC\C(C)=C\C(O)=O)C(=C)CCC2C1(C)C**	**0.560000002**	**A**	**Reliable**
**120**	**Diterpene CM2 14**	**C\C(CC[C@H]1C2CC[C@@H](O)C(C)(C)C2CCC1=C)=C/C(O)=O**	**0.569999993**	**A**	**Reliable**
**121**	**Diterpene CM4 15**	**[H]C1CCC2[C@H](CC\C(C)=C\C(O)=O)C(=C)CCC2C1(C)C**	**0.550000012**	**A**	**Reliable**
**122**	**Diterpene CM7 16**	**[H]C1CCC2[C@H](CC\C(C)=C\C(O)=O)C(=C)CCC2[C@]1(C)C(O)=O**	**0.540000021**	**A**	**Reliable**
**123**	**Diterpene CM8 17**	**C\C(CC[C@H]1C2CC[C@@H](O)[C@@](C)(CO)C2CCC1=C)=C/C(O)=O**	**0.579999983**	**A**	**Reliable**
**124**	**Diterpene CP1 21**	**[H]C1C[C@@]2(C)C(CCC=C2C(O)=O)[C@](C)(CCC2=COC=C2)[C@H]1C**	**0.519999981**	**A**	**Reliable**
**125**	**Diterpene CP3 22**	**C[C@H]1[C@H](C[C@@]2(C)C(CCC=C2C(O)=O)[C@]1(C)CCC1=COC=C1)OC(C<br>)=O**	**0.519999981**	**A**	**Reliable**
**126**	**Diterpene CP2 23**	**[H][C@]12CC=C(C)[C@@H](CC\C(C)=C\C(O)=O)[C@]1(C)CCCC2(C)C**	**0.529999971**	**A**	**Reliable**
**127**	**Diterpene CP4 24**	**[H][C@@]12CCC=C(C(O)=O)[C@@]1(C)CC[C@H](C)[C@@]2(C)CCC(O)CC(O)=<br>O**	**0.529999971**	**A**	**Reliable**

**128**	**Diterpene CT11 25**	**[H][C@@]12CCC=C(C(O)=O)[C@@]1(C)CC[C@H](C)[C@@]2(C)CC\C(CO)=C\C(O)=O**	**0.550000012**	**A**	**Reliable**
**129**	**Diterpene CT12 26**	**[H][C@@]12CCCC(C)(C)C1=CCC(C)[C@H]2CC\C(C)=C\C(O)=O**	**0.540000021**	**A**	**Reliable**
**130**	Caryophyllene alcohol	CC1(C)CC2C1CCC1(C)CCCC2(O)C1	0.540000021	A	Unreliable
**131**	**Labdan dienol acetate**	**[H][C@@]12CC=C(C)[C@H](CC\C(C)=C\COC(C)=C)[C@@]1(C)CCCC2(C)C**	**0.540000021**	**A**	**Reliable**
**132**	**Kaurenoic acid**	**[H][C@]12CC[C@@]34C[C@@H](CCC3[C@]1(C)CCC[C@@]2(C)C(O)=O)C(=C)C4**	**0.550000012**	**A**	**Reliable**
**133**	Camphene Hydrate	CC1(C)C2CCC(C2)C1(C)O	0.529999971	A	Unreliable
**134**	Mentha-1,8-dien-4-ol	CC(=C)[C@@]1(O)CCC(C)=CC1	0.569999993	A	Unreliable
**135**	**Homopodatiline**	**[H][C@]12[C@@H](CC[C@]1(C)CC[C@]1(C)C2CCC2[C@@]3(C)CC[C@H](O)C(C)(C)C3CC[C@@]12C)C(C)=C**	**0.560000002**	**A**	**Reliable**
**136**	**Lupeol**	**[H][C@]12[C@@H](CC[C@]1(C)CC[C@]1(C)[C@]2([H])CC[C@]2([H])C3CC[C@H](O)C(C)(C)[C@]3([H])CC[C@@]12C)C(C)=C**	**0.560000002**	**A**	**Reliable**
**137**	**Lupenone**	**[H][C@]12[C@@H](CC[C@]1(C)CC[C@]1(C)[C@]2([H])CC[C@]2([H])[C@@]3(C)C=CC(=O)C(C)(C)[C@]3([H])CC[C@@]12C)C(C)C**	**0.529999971**	**A**	**Reliable**
**138**	**Acacic acid lactone**	**CC1C23CCC4C(C)(C)C(O)CCC4(C)C2CC=C2C4CC(C)(C)C(O)CC4(C(O)CC132)C(O)=O**	**0.569999993**	**A**	**Reliable**
**139**	P-cymene	CC(C)C1=CC=C(C)C=C1	0.469999999	I	Unreliable
**140**	Artemisia Ketone	CC(C)=CC(=O)C(C)(C)C=C	0.550000012	A	Unreliable
**141**	Cis-linalool oxide	CC(O)(CCC1OC1(C)C)C=C	0.519999981	A	Unreliable
**142**	Beta citral cycle	[H]C(=O)C1=C(C)CCCC1(C)C	0.529999971	A	Unreliable
**143**	**Lynalil acetate**	**CC(C)=CCC[C@@](C)(OC(C)=O)C=C**	**0.50999999**	**A**	**Reliable**
**144**	Carvacrol	CC(C)C1=CC(O)=C(C)C=C1	0.540000021	A	Unreliable
**145**	7-ephi-sequithujene	[H][C@@]12C[C@@]1(CC=C2C)[C@H](C)CCC=C(C)C	0.460000008	I	Unreliable
**146**	β-funebrene	C[C@@H]1CC[C@H]2C(C)(C)[C@@H]3C[C@]12CCC3=C	0.5	A	Unreliable
**147**	**5,9-Undecadien-2-one**	**CC(C)=CCC\C(C)=C\CCC(C)=O**	**0.540000021**	**A**	**Reliable**
**148**	Cis-cadina-1,4-diene	[H][C@@]12CC[C@H](C)CC1=C(CC=C2C)C(C)C	0.50999999	A	Unreliable
**149**	Beta-acoradiene	C[C@H]1CC[C@H](C(C)=C)[C@@]11CCC(C)=CC1	0.469999999	I	Unreliable
**150**	(E)-beta-ionone	[H]\C(=C(\[H])C1=C(C)CCCC1(C)C)C(C)=O	0.540000021	A	Unreliable
**151**	Beta allaskene	C[C@H]1CCC(=C(C)C)[C@]11CCC(C)=CC1	0.479999989	I	Unreliable

**152**	**γ-Bisabolene**	**CC(C)=CCC\C(C)=C1/CCC(C)=CC1**	**0.5**	**A**	**Reliable**
**153**	**Beta-copaen-4-alpha-ol**	**CC(C)[C@]1(O)C2CC2(C)C2CCC(=C)CC12**	**0.550000012**	**A**	**Reliable**
**154**	**Eusdesma-4(15),7-dien-1-b-ol**	**CC(C)C1=CC[C@@]2(C)[C@H](O)CCC(=C)C2C1**	**0.550000012**	**A**	**Reliable**
**155**	**Hexahydrofarnesyl acetone**	**CC(C)CCCC(C)CCCC(C)CCCC(C)=O**	**0.540000021**	**A**	**Reliable**
**156**	**Farnesyl acetone**	**[H]\C(CC\C([H])=C(/C)CCC=C(C)C)=C(\[H])CCC(C)=O**	**0.529999971**	**A**	**Reliable**
**157**	**Phytol**	**[H]\C(CO)=C(\C)CCC[C@H](C)CCC[C@H](C)CCCC(C)C**	**0.569999993**	**A**	**Reliable**
**158**	**Alpha-cadinol**	**[H][C@@]12C=C(C)CC[C@@]1([H])[C@](C)(O)CC[C@H]2C(C)C**	**0.560000002**	**A**	**Reliable**
**159**	**Caryophyllene oxide**	**C[C@@]12CCC[C@H]3[C@H](CC3(C)C)C(=C)C[C@H]1O2**	**0.50999999**	**A**	**Reliable**
**160**	**Cycloart-23-ene-3beta,25-diol**	**[H]\C(C[C@@H](C)[C@@]1([H])CC[C@@]2(C)[C@]3([H])CC[C@]4([H])[C@]5(C[C@@]35CC[C@]12C)CC[C@H](O)C4(C)C)=C(\[H])C(C)(C)O**	**0.560000002**	**A**	**Reliable**
**161**	**Friedelin**	**[H][C@@]12CCC(=O)[C@H](C)[C@@]1(C)CC[C@@]1([H])[C@@]2(C)CC[C@@]2(C)[C@]3([H])CC(C)(C)CC[C@]3(C)CC[C@]12C**	**0.540000021**	**A**	**Reliable**
**162**	**Cys Phytol**	**[H]\C(CO)=C(/C)CCC[C@H](C)CCC[C@H](C)CCCC(C)C**	**0.569999993**	**A**	**Reliable**
**163**	Alpha linalool	CC(=C)CCCC(C)(O)C=C	0.569999993	A	Unreliable
**164**	p-menth-1-em-ol	CC(C)C1CCC(C)=CC1O	0.569999993	A	Unreliable
**165**	Citronelol	C[C@@H](CCO)CCC=C(C)C	0.560000002	A	Unreliable
**166**	**Cassane 1**	**[H][C@@]12CCC(C=C)=C(C)[C@@]1([H])O[C@H](O)C[C@]1([H])C(C)(C)CCC[C@@]21C**	**0.519999981**	**A**	**Reliable**
**167**	**Cassane 2**	**[H]C(=O)C1=C(CC[C@]2([H])[C@]1([H])O[C@H](O)C[C@]1([H])C(C)(C)CCC[C@@]21C)C=C**	**0.540000021**	**A**	**Reliable**
**168**	**(13E)-labd-13-en-3Beta, 8alpha, 15-triol (24)**	**[H][C@]1(CC\C(C)=C\CO)[C@](C)(O)CC[C@@]2([H])C(C)(C)[C@@H](O)CC[C@]12C**	**0.589999974**	**A**	**Reliable**
**169**	**(13E)-3B,8a-dihydroxylabd-13-em-15-oic acid (25)**	**[H][C@]1(CC\C(C)=C\C(O)=O)[C@](C)(O)CC[C@@]2([H])C(C)(C)[C@@H](O)CC[C@]12C**	**0.560000002**	**A**	**Reliable**
**170**	**Acacid acid**	**CC1(C)CC2C(C[C@@H]1O)[C@](O)(C[C@]1(C)C2=CCC2[C@@]3(C)CC[C@H](O)C(C)(C)C3CC[C@@]12C)C(O)=O**	**0.569999993**	**A**	**Reliable**

**171**	**Sapogenin B (27)**	**CC1(C)CC2C3=CCC4[C@@]5(C)CC[C@H](O)C(C)(C)C5CC[C@@]4(C)[C@]3(C)C[C@@H](O)[C@@]22C[C@@H]1CC2=O**	**0.569999993**	**A**	**Reliable**
**172**	**Acacidiol (28)**	**CC1(C)CC2C(C[C@@H]1O)=CC[C@]1(C)C2=CCC2[C@@]3(C)CC[C@H](O)C(C)(C)C3CC[C@@]12C**	**0.579999983**	**A**	**Reliable**
**173**	**Acacigen B (29)**	**[H]\C(C1C\C(CO1)=C\C)=C(\C)C(=O)O[C@H]1C[C@@]2([C@H](O)C[C@]3(C)C(CCC4[C@@]5(C)CC[C@H](O)C(C)(C)C5CC[C@@]34C)C2CC1(C)C)C(O)=O**	**0.550000012**	**A**	**Reliable**
**174**	**(Z) Jasmone**	**[H]\C(CC)=C(/[H])CC1=C(C)CCC1=O**	**0.529999971**	**A**	**Reliable**
**175**	(E)-4,8-dimethyl-1,3,7-nonatriene	[H]\C(C=C)=C(\C)CCC=C(C)C	0.49000001	I	Unreliable
**176**	(Z)-4,8-dimethyl-1,3,7-nonatriene	[H]\C(C=C)=C(/C)CCC=C(C)C	0.49000001	I	Unreliable
**177**	Germacrene A	[H]\C1=C(C)/CC[C@@H](C\C([H])=C(C)\CC1)C(C)=C	0.479999989	I	Reliable
**178**	**Dihydroagathic acid**	**CC(CCC1C(=C)CCC2C1(C)CCCC2(C)C(O)=O)CC(O)=O**	**0.569999993**	**A**	**Reliable**
**179**	**Agathic acid**	**[H]\C(=C(\C)CC[C@H]1C(=C)CC[C@]2([H])[C@]1(C)CCC[C@]2(C)C(O)=O)C(O)=O**	**0.519999981**	**A**	**Reliable**
**180**	**Ent-agathic acid methyl ester**	**[H]\C(=C(\C)CC[C@@H]1C(=C)CC[C@]2([H])C(CCC[C@@]12C)C(=O)OC)C(=O)OC**	**0.550000012**	**A**	**Reliable**
**181**	**Alepterolic acid**	**[H]\C(=C(\C)CC[C@H]1C(=C)CC[C@@]2([H])C(C)(C)[C@@H](O)CC[C@]12C)C(O)=O**	**0.550000012**	**A**	**Reliable**
**182**	**3 beta-hydroxycopalic acid**	**[H]\C(=C(\C)CC[C@@H]1C(=C)CC[C@]2([H])C(C)(C)[C@@H](O)CC[C@@]12C)C(O)=O**	**0.550000012**	**A**	**Reliable**
**183**	**Polyalthic acid**	**[H][C@]12CCC(=C)[C@@H](CCC3=COC=C3)[C@]1(C)CCC[C@]2(C)C(O)=O**	**0.529999971**	**A**	**Reliable**
**184**	**Lambertianic acid**	**C[C@]12CCC[C@@](C)(C1CCC(=C)[C@H]2CCC1=COC=C1)C(O)=O**	**0.529999971**	**A**	**Reliable**
**185**	**Hardwickii acid**	**[H][C@@]12CCC=C(C(O)=O)[C@@]1(C)CC[C@H](C)[C@@]2(C)CCC1=COC=C1**	**0.519999981**	**A**	**Reliable**
**186**	Unreleased compound 01	CC1CC=CC2=C1CCC(C2)=C(C)C	0.449999988	I	Unreliable
**187**	**Unreleased compound 01**	**CCCCCCCCC(O)COC(=O)CCCCCC\C=C\C=C\C=C\CC**	**0.529999971**	**A**	**Reliable**
**188**	Delta-2-carene	[H][C@@]12CCC(C)=C[C@]1([H])C2(C)C	0.460000008	I	Unreliable
**189**	Carvone oxide	[H][C@]12C[C@H](CC(=O)[C@@]1(C)O2)C(C)=C	0.540000021	A	Unreliable

190	Cyclopside 1	C\C(=C/CCC(C)(O[C@H]1OC(CO)[C@@H](OC(C)(CC\C=C(\C)C(O)=O)C=C)[C@@H](O)C1O)C=C)C(O)=O	0.50999999	A	Reliable
191	Ciclopside 2	CC1O[C@H](OC(C)(CC\C=C(/C)C(O)=O)C=C)[C@H](O)C(O)[C@@H]1OC(C)(CC\C=C(/C)C(O)=O)C=C	0.50999999	A	Reliable
192	Compound 175 (S)	[H][C@]1(CC[C@@](C)(O)C=C)[C@](C)(O)CC[C@@]2([H])C(C)(C)CCC[C@]12C	0.560000002	A	Reliable
193	Composto 176 R	[H][C@]1(CC[C@](C)(O)C=C)[C@](C)(O)CC[C@@]2([H])C(C)(C)CCC[C@]12C	0.560000002	A	Reliable
194	Composto 177	[H][C@]1(CC\C(C)=C\CO)[C@](C)(O)CC[C@@]2([H])C(C)(C)CCC[C@]12C	0.579999983	A	Reliable
195	Oleanoic Acid	[H]C1C[C@]2(C)C(=CC[C@]3([H])[C@@]4(C)CC[C@H](O)C(C)(C)[C@]4([H])CC[C@@]23C)[C@]2([H])CC(C)(C)CC[C@]12C(O)=O	0.569999993	A	Reliable
196	Ácido equinocístico	[H][C@@]12CC(C)(C)CC[C@@]1([C@H](O)C[C@]1(C)C2=CC[C@]2([H])[C@@]3(C)CC[C@H](O)C(C)(C)[C@]3([H])CC[C@@]12C)C(O)=O	0.569999993	A	Reliable
197	3,21-dioxo-olean-18-en-oic acid 182	CC1(C)C=C2C3CCC4[C@@]5(C)CCC(=O)C(C)(C)C5CC[C@@]4(C)[C@]3(C)CC[C@]2(CC1=O)C(O)=O	0.560000002	A	Reliable
198	Corosolic acid 183	C[C@@H]1CC[C@@]2(CC[C@]3(C)C(=CCC4[C@@]5(C)C[C@@H](C)[C@H](O)C(C)(C)C5CC[C@@]34C)C2[C@H]1C)C(O)=O	0.579999983	A	Reliable
199	Lupenyl palmitate 186	[H][C@]12[C@@H](CC[C@]1(C)CC[C@]1(C)C2CC[C@]2([H])[C@@]3(C)CC[C@H](OC(=O)CCCCCCCCCCCCCC)C(C)(C)[C@]3([H])OC[C@@]12C)C(C)=C	0.550000012	A	Reliable
200	Lupenyl cynamate 187	[H][C@]12[C@@H](CC[C@]1(C)CC[C@]1(C)C2CC[C@]2([H])[C@@]3(C)CC[C@H](OC(=O)\C=C\C4=CC=CC=C4)C(C)(C)[C@]3([H])OC[C@@]12C)C(C)=C	0.519999981	A	Reliable
201	Alpha amyrin	[H][C@]12CC=C3[O@]4([H])CC(C)(C)CC[O@]4(C)CC[C@@]3(C)[C@]1(C)CCC1C(C)(C)[C@H](CC[C@]21C)OC(=O)\C=C/C1=CC=C(O)C=C1	0.5	A	Reliable
202	Beta- amyrin	[H][C@@]12CC(C)(C)CC[C@]1(C)CC[C@]1(C)C2=CC[C@]2([H])[C@@]3(C)CC[C@@H](OC(=O)\C=C\C4=CC=C(O)C=C4)C(C)(C)C3CC[C@@]12C	0.529999971	A	Reliable
203	Trans-hydroxycinnamoyl ester of lupeol	[H][C@]12CCC3C4[C@@H](CC[C@]4(C)CC[C@@]3(C)[C@]1(C)CCC1C(C)(C)[C@H](CC[C@]21C)OC(=O)\C=C\C1=CC=C(O)C=C1)C(C)=C	0.519999981	A	Reliable
204	erythrodiol	CC1(C)CC[C@]2(CO)CC[C@]3(C)C(=CCC4[C@@]5(C)CCC(O)C(C)(C)C5CC[C@@]34C)C2C1	0.589999974	A	Reliable

**205**	**3-b-o- trans-p-coumaroyl-erythrodiol**	**CC1(C)CC[C@]2(CO)CC[C@]3(C)C(=CCC4[C@@]5(C)CCC(OC(=O)C=CC6=CC=C(O)C=C6)C(C)(C)C5CC[C@@]34C)C2C1**	**0.540000021**	**A**	**Reliable**
**206**	**Dihidroespinasterol**	**CCC(CCC(C)C1CCC2C3=CCC4CC(O)CCC4(C)C3CCC12C)C(C)C**	**0.579999983**	**A**	**Reliable**
**207**	**Campestenone**	**CC(C)C(C)CCC(C)C1CCC2C3CC=C4CC(=O)CCC4(C)C3CCC12C**	**0.540000021**	**A**	**Reliable**
**208**	**19-O-trans-feruloyl-labd-8(17)**	**C[C@H](CCO)CC[C@@H]1C(=C)CCC2[C@](C)(COC(=O)\C=C\C3=CC=C(O)C(O)=C3)CCC[C@@]12C**	**0.540000021**	**A**	**Reliable**
**209**	**19-O-[(E)-30 ,40 -dimethoxy cinnamoyl]-labd-8(17)-en- 15,19-diol**	**COC1=CC=C(\C=C\C(=O)OC[C@]2(C)CCC[C@@]3(C)[C@H](CC[C@H](C)CCO)C(=C)CCC23)C=C1O**	**0.550000012**	**A**	**Reliable**
**210**	**Querataroic acid**	**[H][C@@]12C[C@@](C)(CO)CC[C@@]1(CC[C@]1(C)C2=CC[C@]2([H])[C@@]3(C)CC[C@H](O)C(C)(C)[C@]3([H])CC[C@@]12C)C(O)=O**	**0.579999983**	**A**	**Reliable**
**211**	**Hederagenim**	**[H][C@@]12CC(C)(C)CC[C@@]1(CC[C@]1(C)C2=CC[C@]2([H])[C@@]3(C)CC[C@H](O)[C@@](C)(CO)[C@]3([H])CC[C@@]12C)C(O)=O**	**0.579999983**	**A**	**Reliable**
**212**	**Bayogenin**	**[H][C@@]12CC(C)(C)CC[C@@]1(CC[C@]1(C)C2=CC[C@]2([H])[C@@]3(C)C[C@H](O)[C@H](O)[C@@](C)(CO)[C@]3([H])CC[C@@]12C)C(O)=O**	**0.569999993**	**A**	**Reliable**
**213**	Medicagenic acid	[H][C@@]12CC(C)(C)CC[C@@]1(CC[C@]1(C)C2=CC[C@]2([H])[C@@]3(C)C[C@H](O)[C@H](O)[C@@](C)(C(O)=O)[C@]3([H])CC[C@@]12C)C(O)=O	0.569999993	A	Unreliable
**214**	Zanhic acid	[H][C@@]12CC[C@]3(C)[C@]([H])(CC=C4C5CC(C)(C)CC[C@@]5([C@@H](O)C[C@@]34C)C(O)=O)[C@@]1(C)C[C@H](O)[C@H](O)[C@@]2(C)C(O)=O	0.560000002	A	Unreliable
**215**	**Melilotiogenin metil ester**	**COC(=O)[C@@]1(C)CC2C3CCC4[C@@]5(C)CC[C@H](O)[C@](C)(CO)C5CC[C@@]4(C)[C@]3(C)CC[C@@]2(C)C(=O)C1**	**0.589999974**	**A**	**Reliable**
**216**	**Kaur-16-ene**	**CC1(C)CCCC2(C)C3CCC4CC3(CC4=C)CCC12**	**0.529999971**	**A**	**Reliable**
**217**	**Triterpenoid**	**[H][C@@]12CC(C)(C)CC[C@@]1(CC[C@]1(C)C2=CC[C@]2([H])[C@@]3(C)CC[C@H](O)[C@@](C)(COS(O)(=O)=O)[C@]3([H])CC[C@@]12C)C(O)=O**	**0.529999971**	**A**	**Reliable**

Subtitle: A (Active compounds) and I (Inactive Compounds).

(I)

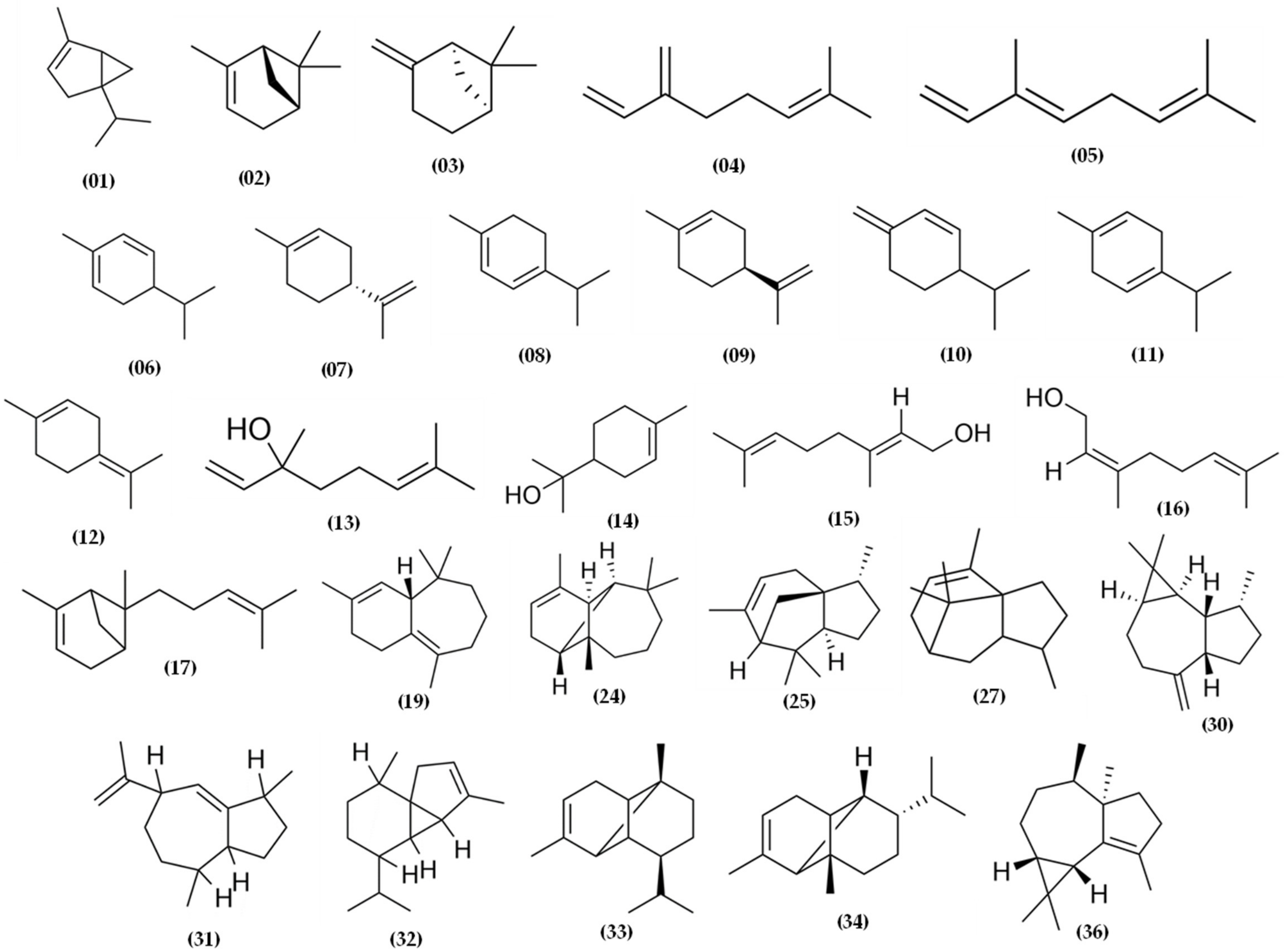
(01)
(02)
(03)
(04)
(05)
(06)
(07)
(08)
(09)
(10)
(11)
(12)
(13)
(14)
(15)
(16)
(17)
(19)
(24)
(25)
(27)
(30)
(31)
(32)
(33)
(34)
(36)
HO
OH
H

(II)

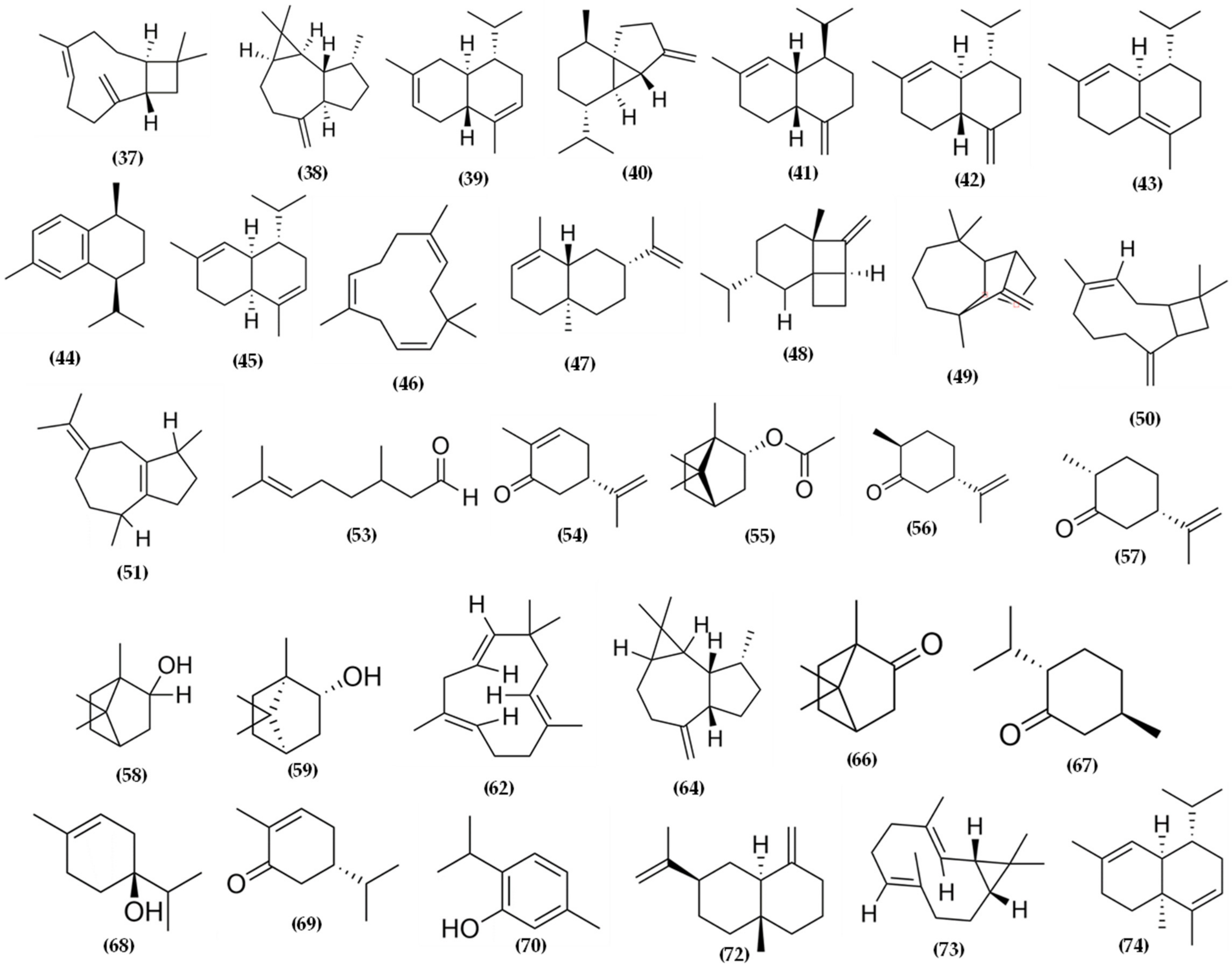
(37)
(38)
(39)
(40)
(41)
(42)
(43)
(44)
(45)
(46)
(47)
(48)
(49)
(50)
(51)
(53)
(54)
(55)
(56)
(57)
(58)
(59)
(62)
(64)
(66)
(67)
(68)
(69)
(70)
(72)
(73)
(74)

(III)

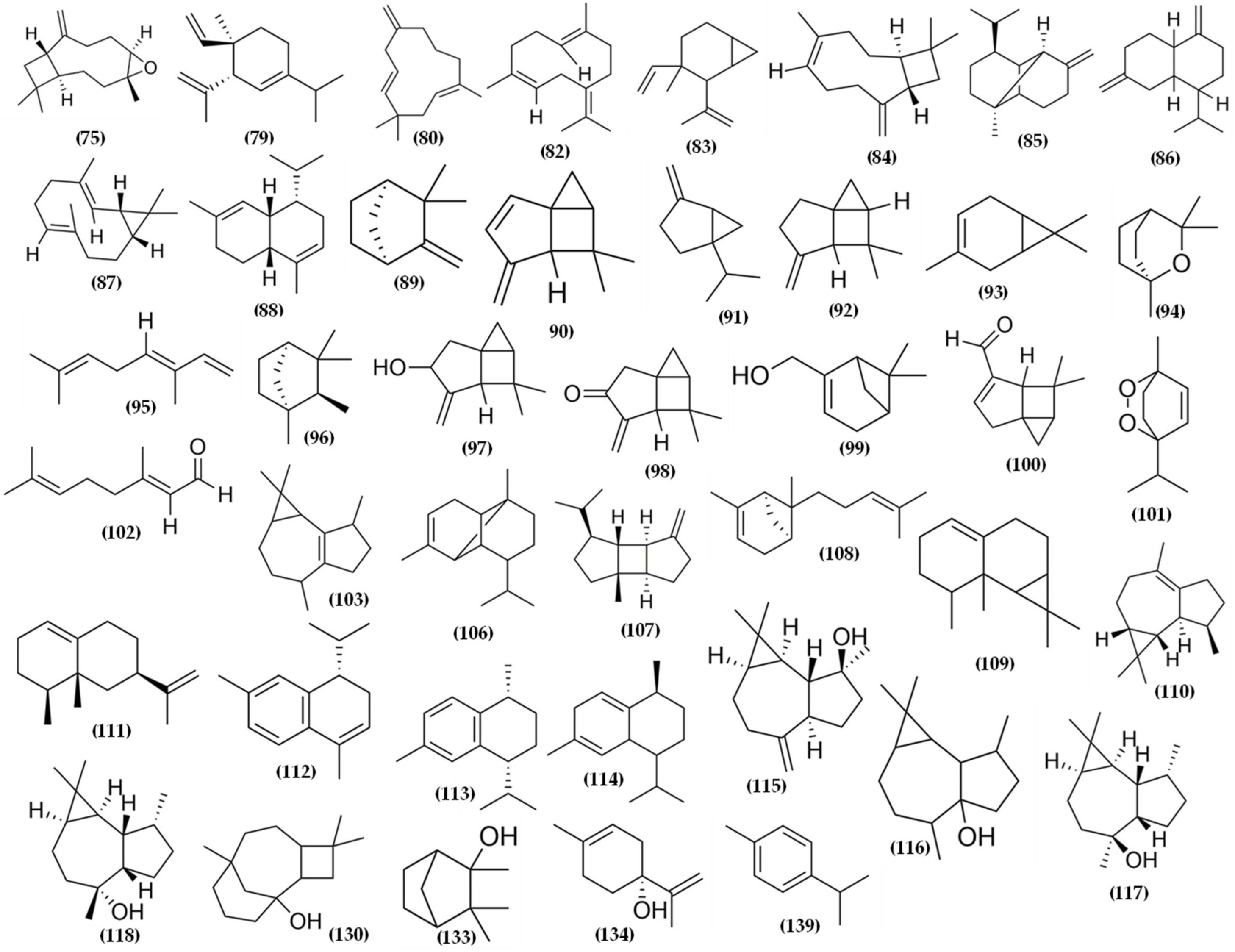
(75)
(79)
(80)
(82)
(83)
(84)
(85)
(86)
(87)
(88)
(89)
90)
(91)
(92)
(93)
(94)
(95)
(96)
(97)
(98)
(99)
(100)
(101)
(102)
(103)
(106)
(107)
(108)
(109)
(110)
(111)
(112)
(113)
(114)
(115)
(116)
(117)
(118)
(130)
(133)
(134)
(139)

(IV)

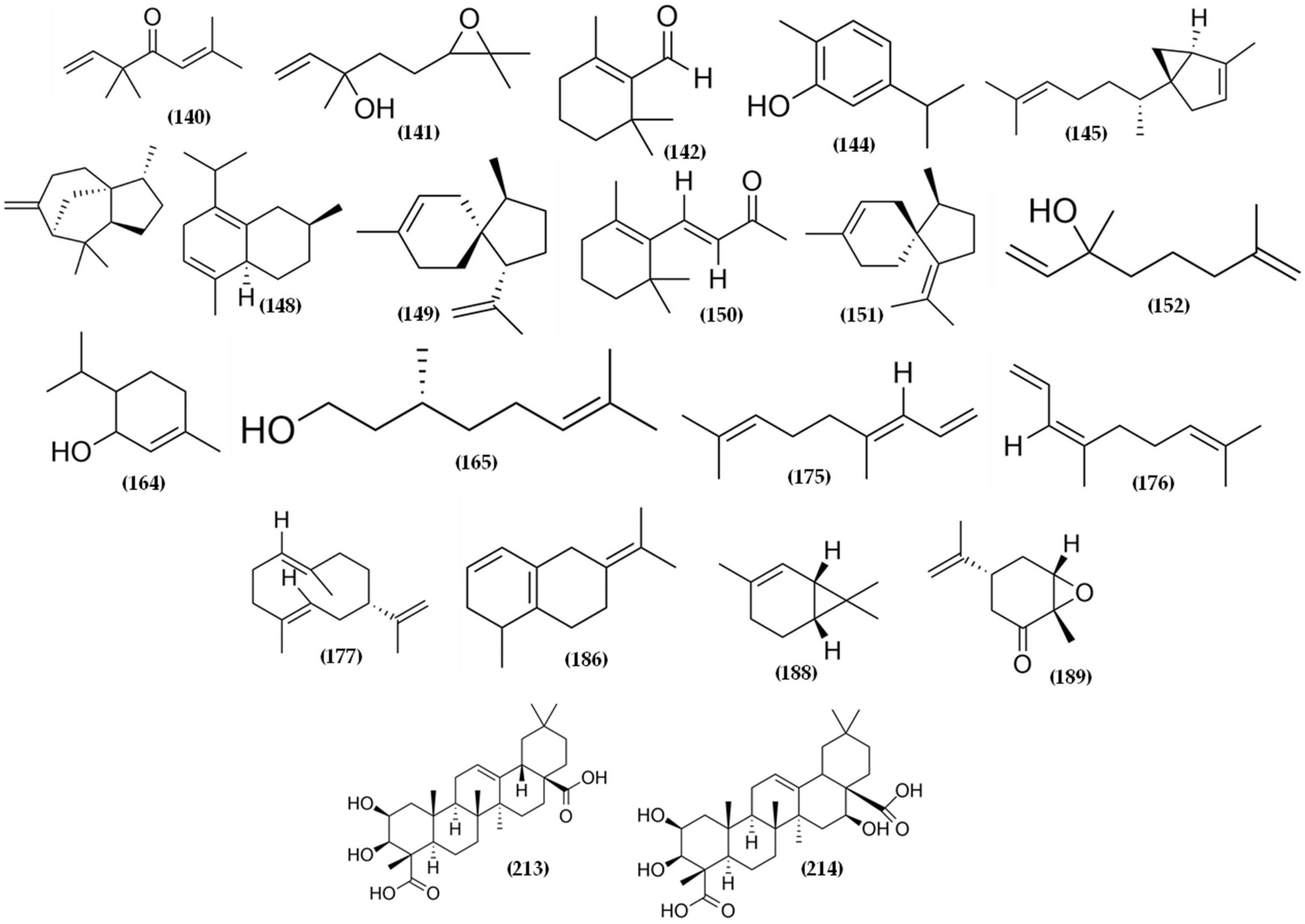

Figure S1: Compounds that did not show reliability in the applicability domain in the model created with AlvaDesc descriptors.

(I)

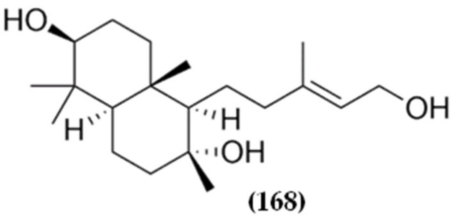

(168)
p=0.589

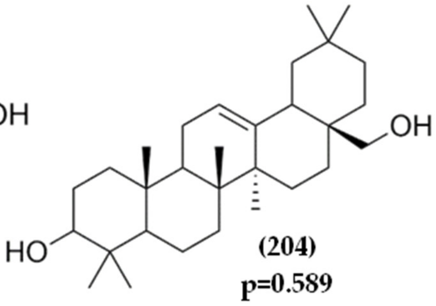

(204)
p=0.589

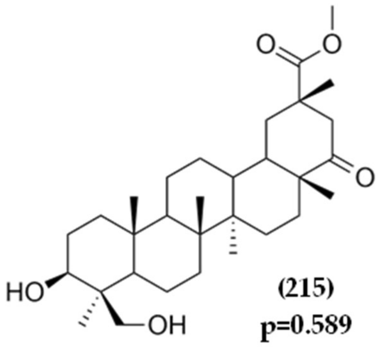

(215)
p=0.589

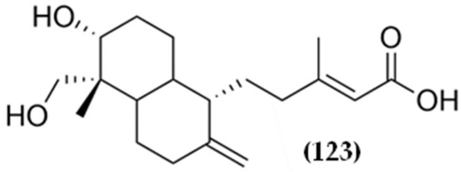

(123)
p=0.579

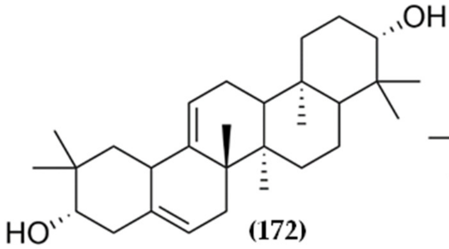

(172)
p=0.579

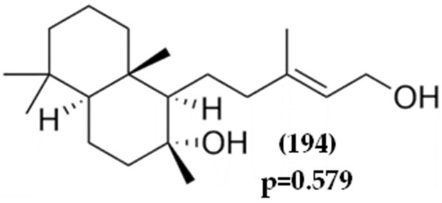

(194)
p=0.579

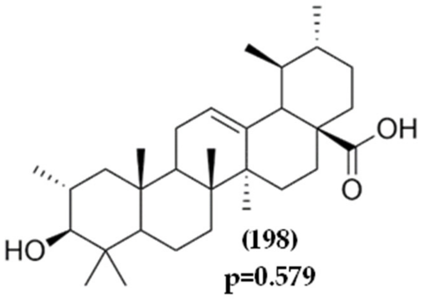

(198)
p=0.579

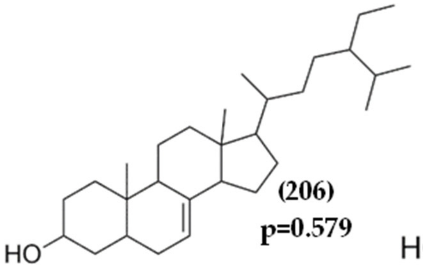

(206)
p=0.579

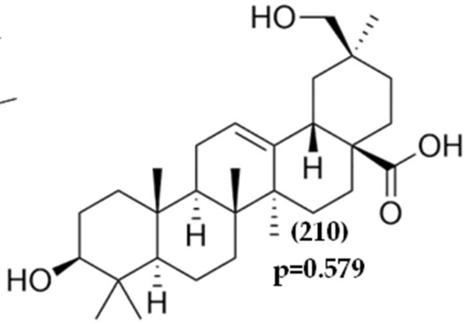

(210)
p=0.579

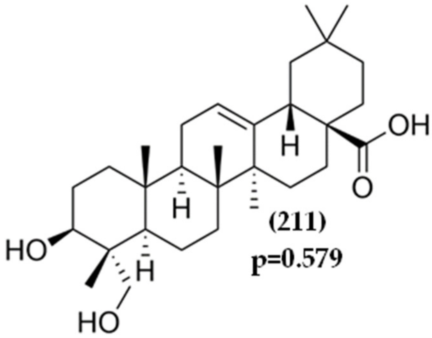

(211)
p=0.579

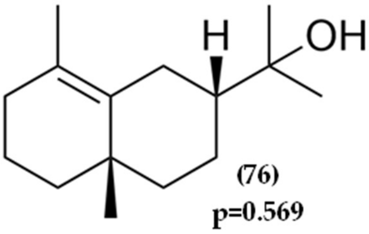

(76)
p=0.569

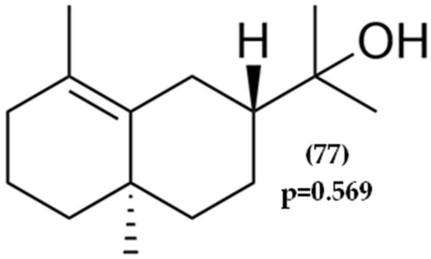

(77)
p=0.569

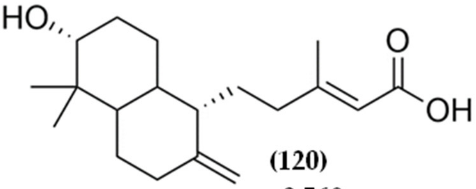

(120)
p=0.569

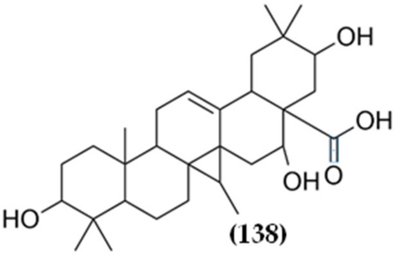

(138)
p=0.569

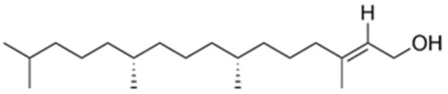

(157)
p=0.569

(II)

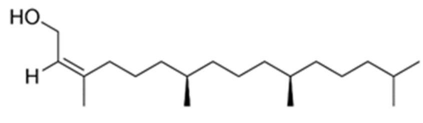

(162)
p=0.569

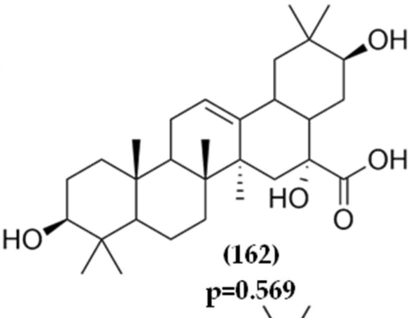

(162)
p=0.569

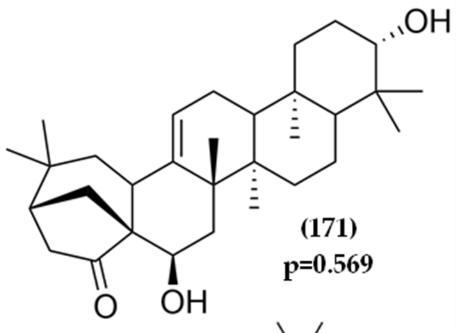

(171)
p=0.569

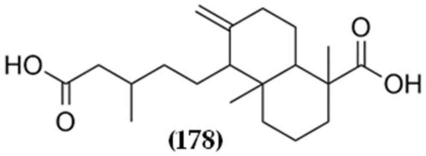

(178)
p=0.569

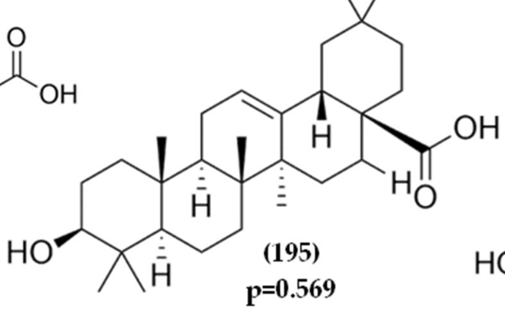

(195)
p=0.569

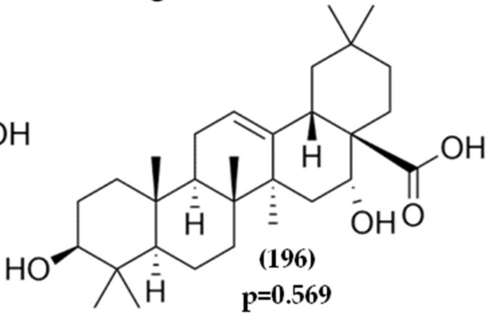

(196)
p=0.569

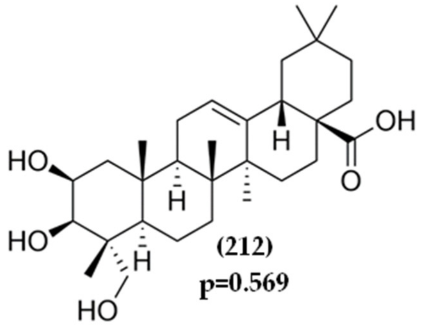

(212)
p=0.569

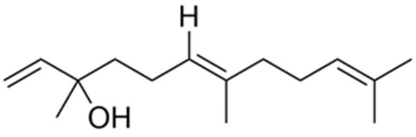

(28)
p=0.560

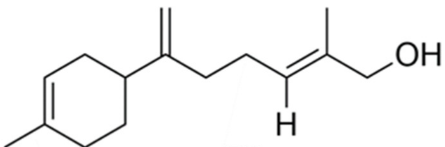

(52)
p=0.560

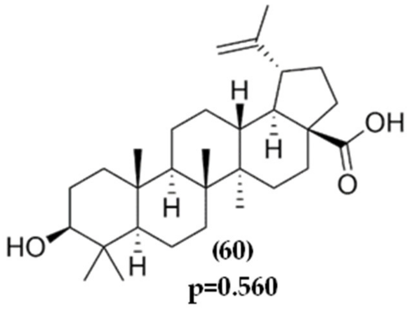

(60)
p=0.560

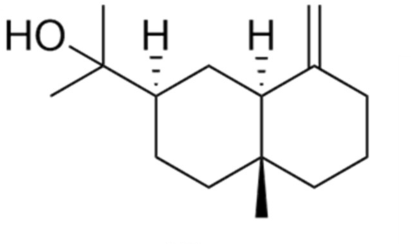

(78)
p=0.560

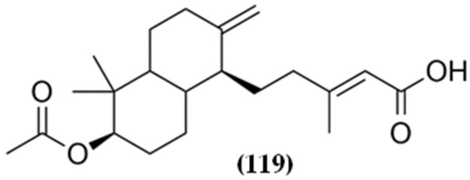

(119)
p=0.560

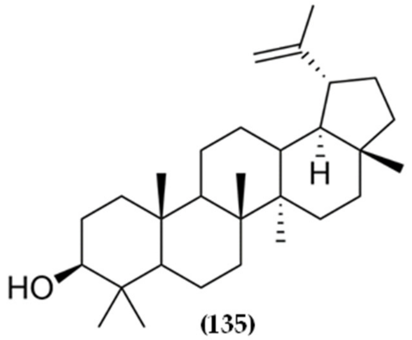

(135)
p=0.560

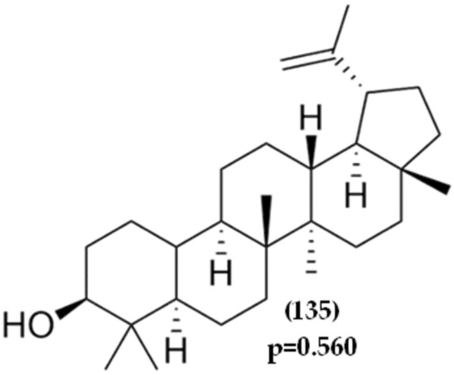

(135)
p=0.560

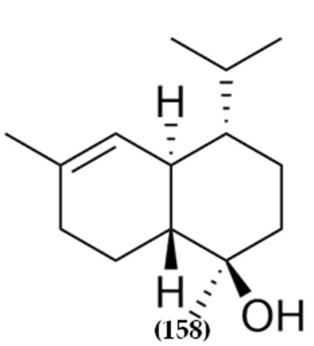

(158)
p=0.560

(III)

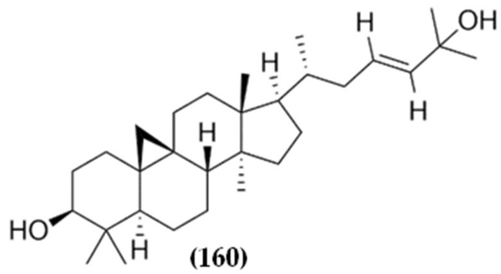

(160)
p=0.560

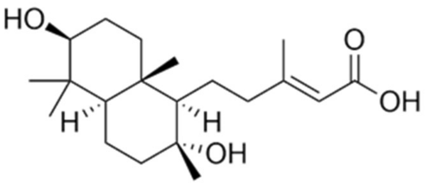

(169)
p=0.560

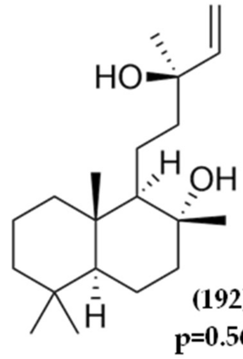

(192)
p=0.560

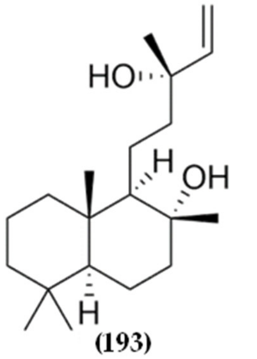

(193)
p=0.560

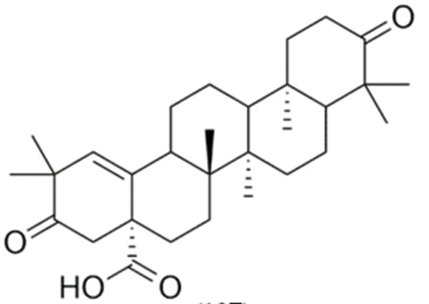

(197)
p=0.560

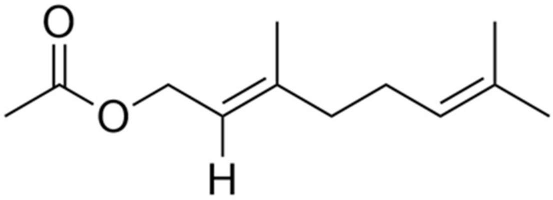

(104)
p=0.550

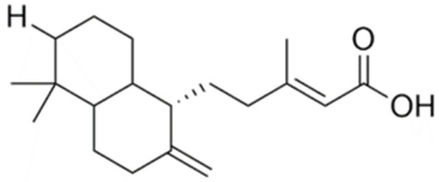

(121)
p=0.550

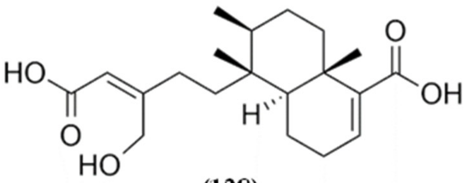

(128)
p=0.550

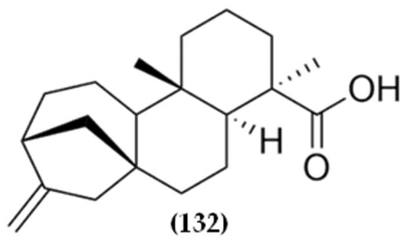

(132)
p=0.550

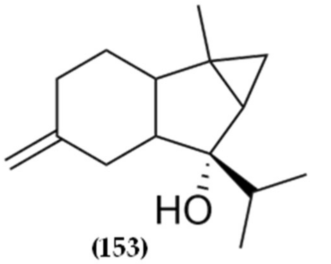

(153)
p=0.550

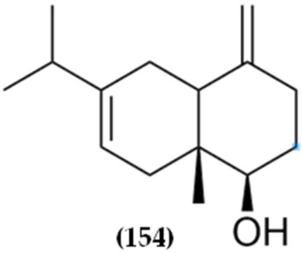

(154)
p=0.550

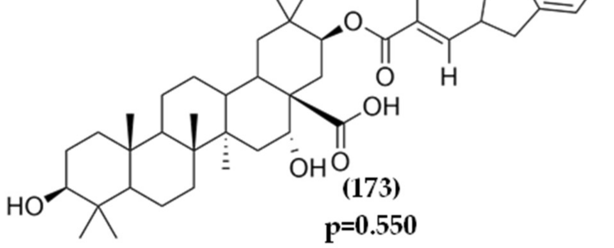

(173)
p=0.550

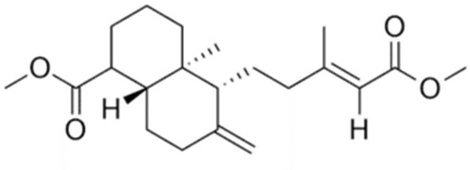

(180)
p=0.550

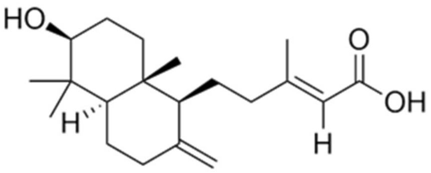

(181)
p=0.550

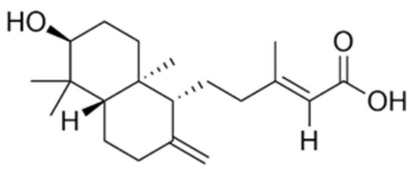

(182)
p=0.550

(IV)

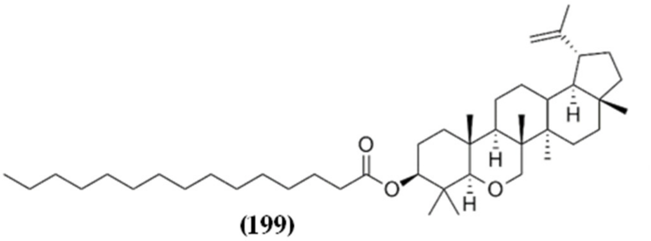

(199)
p=0.550

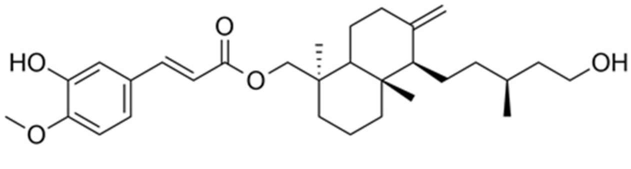

(209)
p=0.550

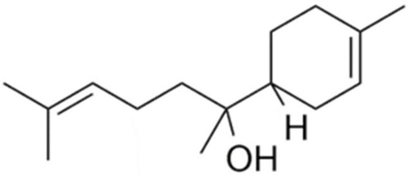

(29)
p=0.540

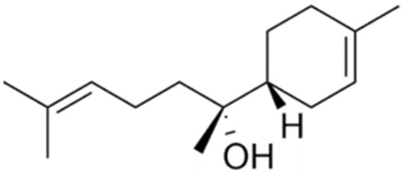

(63)
p=0.540

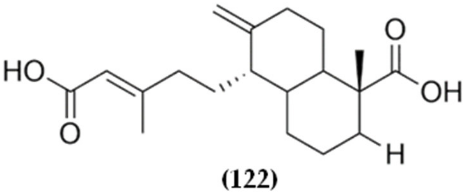

(122)
p=0.540

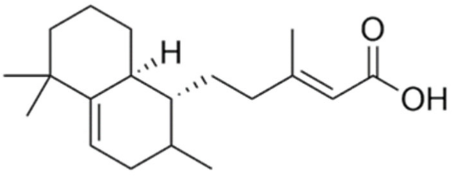

(129)
p=0.540

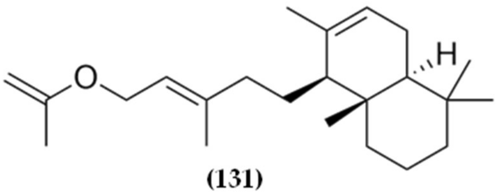

(131)
p=0.540

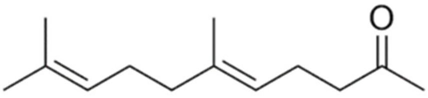

(147)
p=0.540

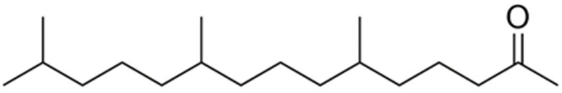

(155)
p=0.540

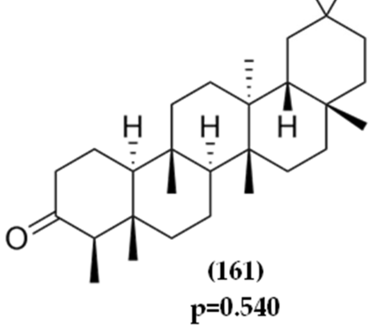

(161)
p=0.540

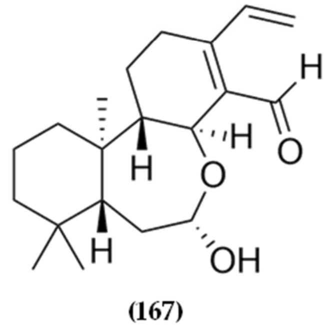

(167)
p=0.540

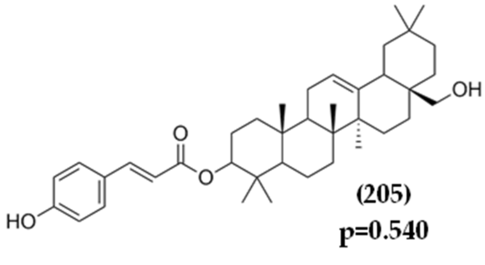

(205)
p=0.540

(V)

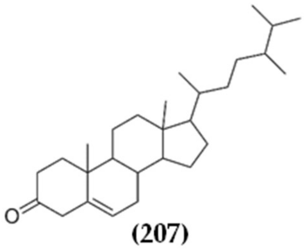

(207)
p=0.540

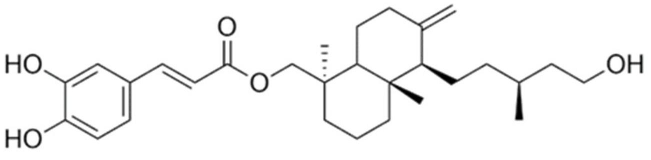

(208)
p=0.540

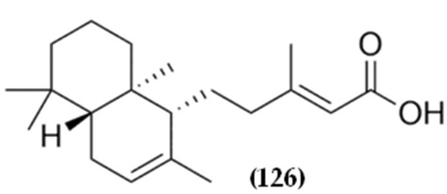

(126)
p=0.529

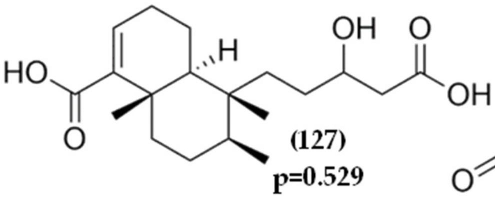

(127)
p=0.529

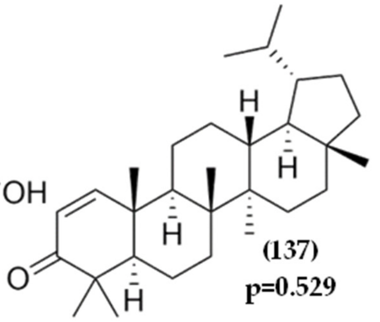

(137)
p=0.529

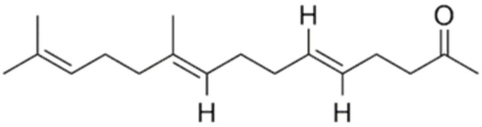

(156)
p=0.529

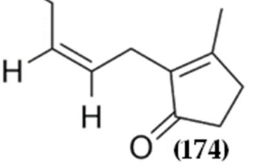

(174)
p=0.529

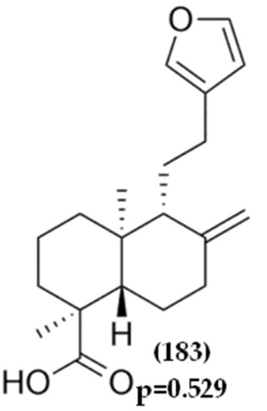

(183)
p=0.529

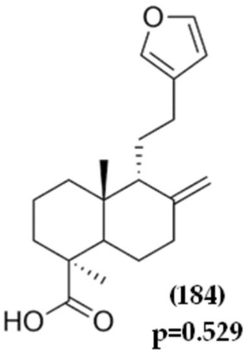

(184)
p=0.529

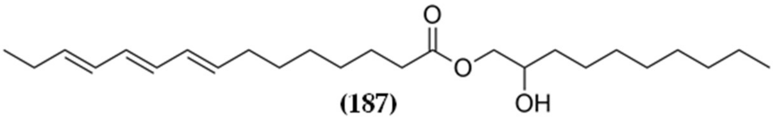

(187)
p=0.529

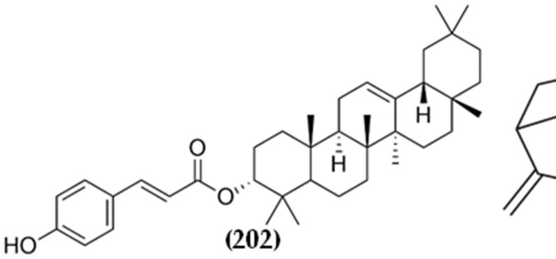

(202)
p=0.529

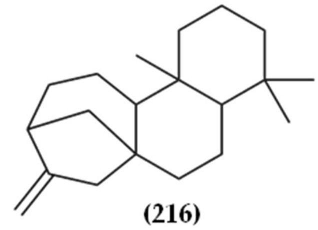

(216)
p=0.529

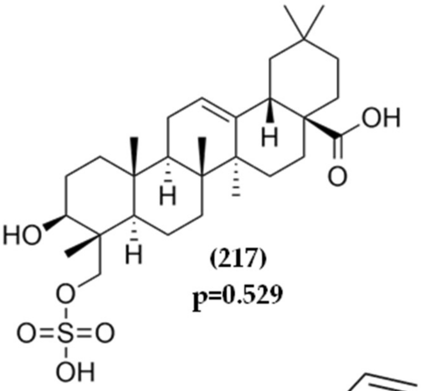

(217)
p=0.529

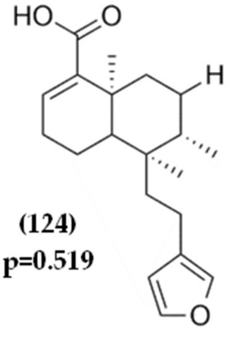

(124)
p=0.519

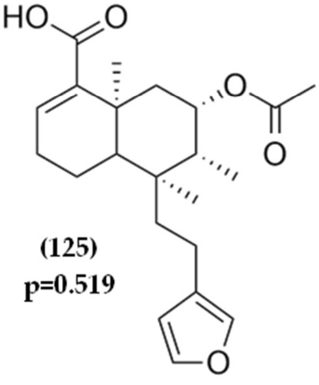

(125)
p=0.519

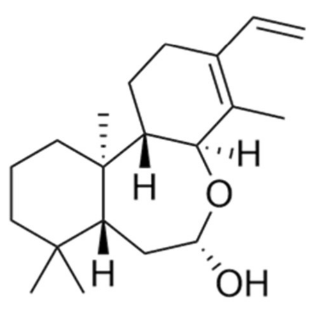

(166)
p=0.519

(VI)

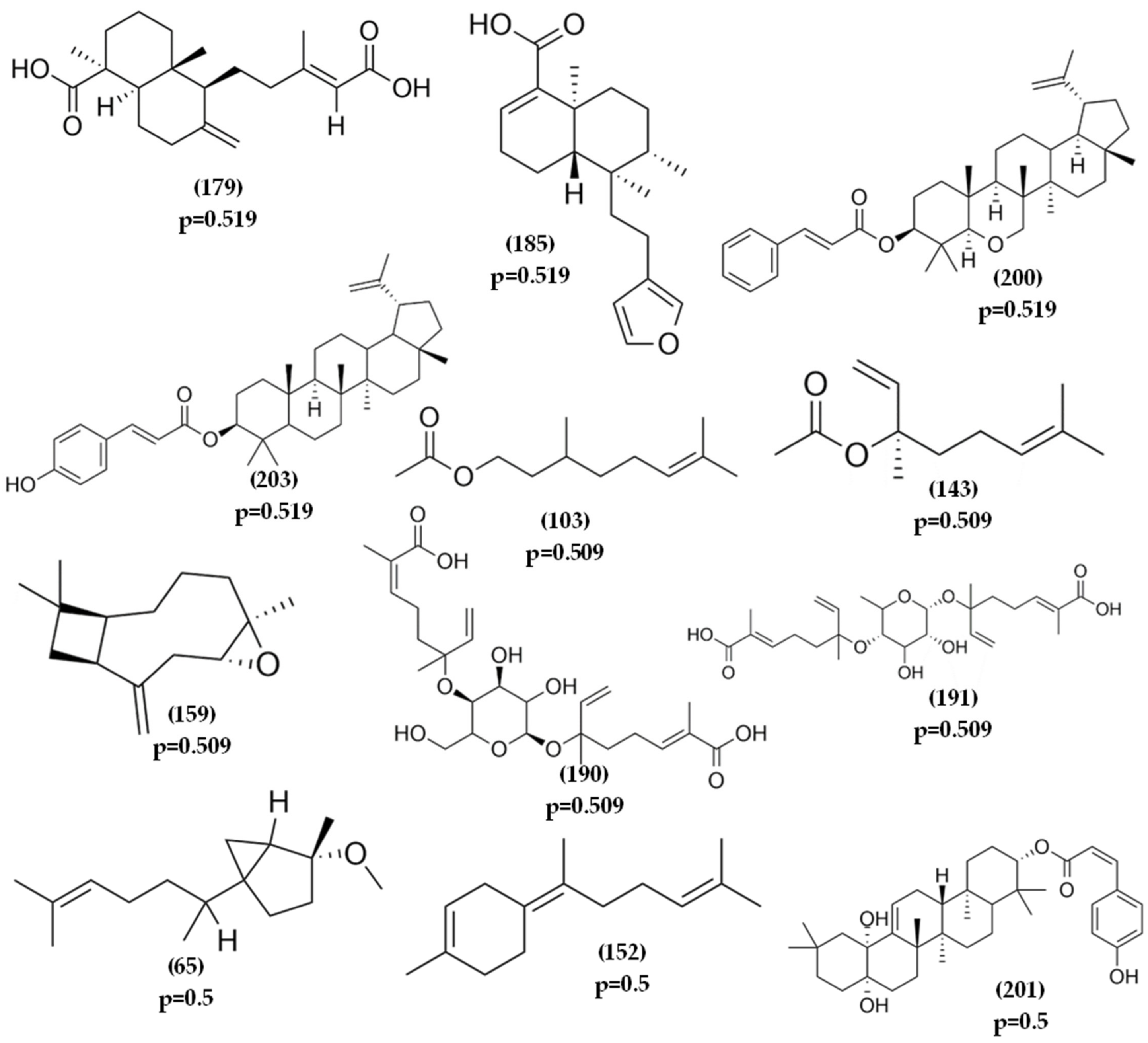

Figure S2: Compounds that presented activity probability values above 0.50 and reliable applicability domain in the prediction model created with AlvaDesc descriptors.

1. Molecular Docking

Observing the value (best score) of RMSD is a good way to evaluate the ability of a method to find the binding mode of a ligand in a set of positions. An RMSD value less than 2.0 Å is considered reliable for reproducing a known binding mode, indicating the success of the method [60,61]. The redocking, including the RMSD (Root Mean Square Deviation) values which measure the deviation between the experimentally determined crystallographic structure and the coupled pose, are shown in Table S3. [59,60]. The proteins subjected to redocking calculations were Main-protease (M-pro) (5RG1) and Papain-like protease (PL-pro) (PDB: 7TZJ), which have a co-crystallized ligand, and Spike protein (PDB: 8C8P) and RNA-dependent RNA polymerase (PDB: 6M71), whose coordinates were determined by the Bite Net platform - Skolteck I Molecule, 2023 (https://sites.skoltech.ru/imolecule/tools/bitenet). The active site region corresponds to the amino acids mentioned in the PDB reference article for subunit (A), with corresponding coordinates

Center: A. The RMSD values for the ligand and redocking using the MolDock Score algorithm can be found in Table 4.

Table S3. RMSD values for the protein selected in the study.

Protein	Ligand PDB ID	RMSD
Main-Protease (M-pro) (PDB: 5RG1)	Nalpha-acetyl-N-(3-bromoprop-2-yn-1-yl)-L-tyrosinamide (T9J)	1.92
Papain-Like protease (PL-pro) (PDB: 7TZJ)	N-[(3-fluorophenyl)methyl]-1-[(1R)-1-naphthalen-1-ylethyl]piperidine-4-carboxamide (S88)	0.23

In the redocking analysis, it was observed that the RMSD values were below 2.0 Å, indicating that the generated poses correctly positioned the ligands in the active site (Figure S3). This suggests that the program provided satisfactory values for docking validation.

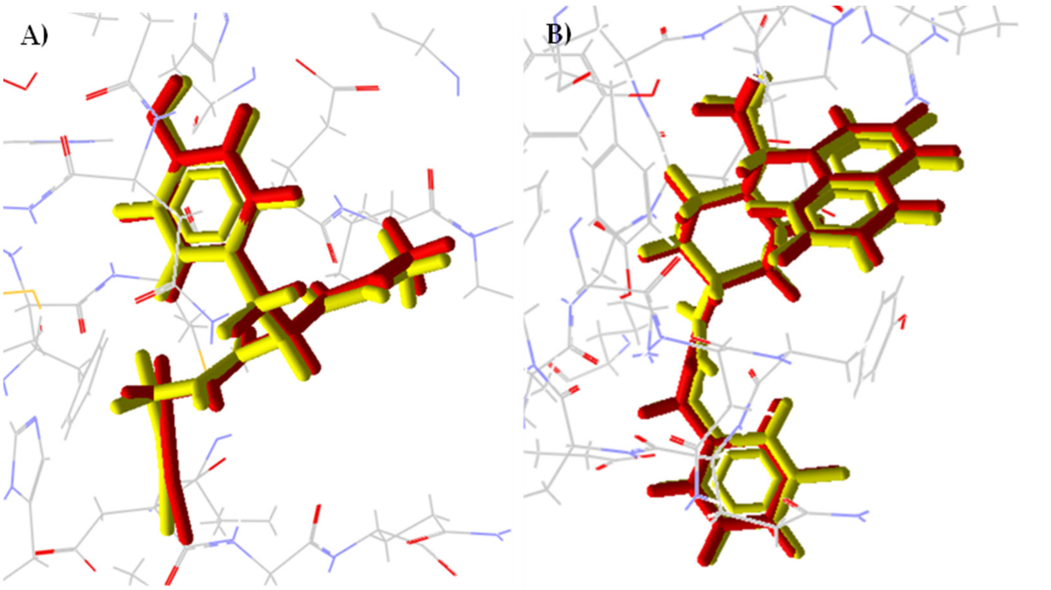

Figure S3. Redocking of the co-crystallized ligands and their respective poses. A) M-protease target (PDB: 5RG1) and Papain-like protease target (PDB: 7TZJ).

Legend: Yellow (most stable pose of the ligand) and red (ligand).

For the Papain-like protease (PDB: 7TZJ), the PDB ligand exhibited the highest affinity at −123.07 KJ·mol^{-1} (p = 1). The test compounds presented −97.57 KJ·mol^{-1} (p = 0.79) for betulinic acid. Regarding the Spike protein (PDB: 8C8P), the most stable compound was the positive control Nirmatrelvir at −149.45 KJ·mol^{-1} (p = 1). The test compounds exhibited a probability of activity greater than 0.6, with −102.35 KJ·mol^{-1} (p = 0.68) for Betulinic acid and −96.91 KJ·mol^{-1} (p = 0.64) for Lupeol.

For the enzyme RNA-dependent RNA polymerase (PDB: 6M71), the compounds under study did not present probability values above 0.5, corresponding to 0.46 for Lupeol and 0.45 for Betulinic Acid, with the control remdesivir being the compound with the highest probability 1 Thus, it can be seen that the target for which the compounds showed greater affinity corresponded to Main-Protease (PDB: 5RG1).

Figure S2 illustrates the interaction of the compounds: Lupeol (A), Betulinic acid (B), the control drug and the PDB Ligand (C) with the target Main-protease (M-pro) (PDB: 5RG1). In Figure 3, the molecular interactions with the Main-protease (M-pro) (PDB: 5RG1) involved hydrogen bonds (green dashed lines), hydrophobic interactions (pink, blue and orange dashed lines) and steric interactions (red dashed lines).

For Lupeol (A), the interactions with the target ocurred through carbon atoms (C) of the rings that in its chemical structure. The compound interacted with methyl groups

(CH3), indicating a significant contribution of hydrophobic and nonpolar characteristics in the interaction with the enzyme's active site. The residues involved comprised Cys 145 (1 interaction), His 163 (2 interactions), His 172 (1 interaction), Phe 140 (1 interaction), Met 165 (2 interactions), His 41 (1 interaction), Met 49 (3 interactions) and Arg 188 (1 interaction). These interactions were associated with alkyl and pi-alkyl interactions. Alkyl-type interactions involve a group of electrons from the Alkyl group, generating hydrophobic bonds [62], while pi-alkyl interactions indicate an interaction of the electron cloud over an aromatic group and a group of electrons from any Alkyl group, generating hydrophobic bonds [63,64]. Unfavorable interactions (red dashed lines) were observed through residues Gln 189 (1 interaction) and Met 49 (2 interactions).

The interaction of Betulinic acid with the Main-protease (M-pro) (PDB: 5RG1) target demonstrated favorable interactions with carbon atoms (C) from the rings of the chemical structure, imparting a hydrophobic and nonpolar character. These interactions involvede residues Pro168 (2 interactions), His 172 (1 interaction), His 163 (2 interactions) and Cys 145 (1 interaction), and they were categorized as alkyl and pi-alkyl types (pink dashed lines). Adittionally, the carboxylic acid group in betulinic acid exhibited hydrogen bond-type interactions (green dashed lines) with residues Met 165 (1 interaction), Glu 166 (1 interaction), His 164 (1 interaction), His 41 (1 interaction), Cys 145 (1 interaction).

2. Molecular Dynamics

For the Main-protease (M-pro) (PDB: 5RG1) enzyme, Figure S3 showed that the stabilization of the simulation (black line) occurred only at the beginning of the simulation (0 ns to 10 ns) with RMSD values corresponding to 0.3 nm. Subsequently, fluctuations occurred from 20 ns to 40 ns with RMSD values reaching 0.45 nm and 0.5 nm, respectively. The enzyme then returnined to a stable state with RMSD values around 0.3 nm from 0.35 nm to the end of the simulation.

Concerning the test compound Lupeol (green line), stabilization occurred from 10 ns to 55 ns with RMSD values of 0.4 nm. After 55 ns, there was a fluctuation in RMSD values, reaching 0.5 nm around 60 ns. However, Lupeon regained stability after the 80 ns, when the RMSD value dropped to 0.35 nm, remaining constant until the end of the simulation. Lupeol exhibited high stability throughout most of the simulation.

On the other hand, Betulinic acid (red line), showed greater fluctuations in RMSD values compared to Lupeol. The RMSD values for Betulinic acid corresponded to 4.5 nm (from 25 ns to 70 ns), and then dropping to 0.57 nm at 80 ns.

The PDB ligand (blue line), exhibited lower RMSD values, indicating greater stability compared to the test compounds Lupeol and Betulinic acid. The PDB ligand presented RMSD values of up to 4.5 nm and demonstrated stability during the simulation period from 40 ns to 80 ns. The stability of the Main-protease (M-pro) protein (PDB: 5RG1) is essential for maintaining the binding ofcompounds to the active site.

References

1. Bao, T.; Shadrack, K.; Yang, S.; Xue, X.; Li, S.; Wang, N.; Wang, Q.; Wang, L.; Gao, X.; Cronk, Q. Functional Characterization of Terpene Synthases Accounting for the Volatilized-Terpene Heterogeneity in Lathyrus Odoratus Cultivar Flowers. *Plant Cell Physiol.* **2020**, *61*, 1733–1749.
2. Shams-Ardakani, M.; Ghannadi, A.; Badr, P.; Mohagheghzadeh, A. Biotransformation of Terpenes and Related Compounds by Suspension Culture of Glycyrrhiza Glabra L.(Papilionaceae). *Flavour Fragr. J.* **2005**, *20*, 141–144.
3. Silva, V.N.T.; de Oliveira, F.M.; Conserva, L.M. Phenolic Derivatives and Terpenes from Acosmium Bijugum. *Biochem. Syst. Ecol.* **2001**, *29*, 1189–1192.
4. Vila Verde, G.M.; A. Barros, D.; Oliveira, M.S.; Aquino, G.L.B.; M. Santos, D.; De Paula, J.R.; Dias, L.D.; Piñeiro, M.; M. Pereira, M. A Green Protocol for Microwave-Assisted Extraction of Volatile Oil Terpenes from Pterodon Emarginatus Vogel.(Fabaceae). *Molecules* **2018**, *23*, 651.
5. Khallouki, F.; Younos, C.; Soulimani, R.; Bessiere, J.M. Chemical Composition of the Essential Oil of Ononis Natrix L. Fabaceae. *J. Essent. Oil Res.* **2002**, *14*, 431–432.
6. Marinho, C.R.; Martucci, M.E.P.; Gobbo-Neto, L.; Teixeira, S.P. Chemical Composition and Secretion Biology of the Floral Bouquet in Legume Trees (Fabaceae). *Bot. J. Linn. Soc.* **2018**, *187*, 5–25.
7. Egigu, M.C.; Ibrahim, M.A.; Yahya, A.; Holopainen, J.K. Cordeauxia Edulis and Rhododendron Tomentosum Extracts Disturb Orientation and Feeding Behavior of Hylobius Abietis and Phyllodecta Laticollis. *Entomol. Exp. Appl.* **2011**, *138*, 162–174.
8. Carneiro, L.J.; Tasso, T.O.; Santos, M.F.C.; Goulart, M.O.; Santos, R.A. dos; Bastos, J.K.; da Silva, J.J.M.; Crotti, A.E.M.; Parreira, R.L.T.; Orenha, R.P. Copaifera Multijuga, Copaifera Pubiflora and Copaifera Trapezifolia Oleoresins: Chemical Characterization and in Vitro Cytotoxic Potential against Tumoral Cell Lines. *J. Braz. Chem. Soc.* **2020**, *31*, 1679–1689.
9. Saldanha, K.L.A.E.; de Andrade Royo, V.; Da Fonseca, F.S.A.; Menezes, E.V.; De Oliveira, D.A.; de Melo Júnior, A.F.; Teixeira, K.S.; Brandão, M.M.; Leite, M.N.; Caetano, G.F. Cytotoxic Action and Proliferation in Vitro and Analgesic Activity in Vivo of Resin from Hymenaea Stigonocarpa. *Pharmacognosy Res.* **2019**, *11*.
10. Lemos, M.; Santin, J.R.; Mizuno, C.S.; Boeing, T.; Sousa, J.P.B. de; Nanayakkara, D.; Bastos, J.K.; Andrade, S.F. de Copaifera Langsdorffii: Evaluation of Potential Gastroprotective of Extract and Isolated Compounds Obtained from Leaves. *Rev. Bras. Farmacogn.* **2015**, *25*, 238–245.
11. Bhakshu, L.M.; Raju, R.R.V. Chemical Composition and in Vitro Antimicrobial Activity of Essential Oil of Rhynchosia Heynei, an Endemic Medicinal Plant from Eastern Ghats of India. *Pharm. Biol.* **2009**, *47*, 1067–1070.
12. Sousa Júnior, P.T.; Dall'Oglio, E.L.; Silva, L.E. da; Figueiredo, U.S.; Vieira, P.C.; Machado, H. V; Santos, L.G. dos Gênero Acosmium: Composição Química e Potencial Farmacológico. *Rev. Bras. Farmacogn.* **2009**, *19*, 150–157.
13. Omara, T.; Kiprop, A.K.; Kosgei, V.J. Albizia Coriaria Welw Ex Oliver: A Review of Its Ethnobotany, Phytochemistry and Ethnopharmacology. *Adv. Tradit. Med.* **2023**, *23*, 631–646.
14. Ojah, E.O.; Moronkola, D.O.; Ajiboye, C.O.; Yusuf, T.L.; Adeniyi-Akee, M.A. Chemical Compositions, Antioxidant and Anti-Diabetic Activities of Root Wood and Root Bark Essential Oils from Pterocarpus Soyauxii TAUB. *J. Essent. Oil Bear. Plants* **2021**, *24*, 53–67.
15. Al Muqarrabun, L.M.R.; Ahmat, N.; Ruzaina, S.A.S.; Ismail, N.H.; Sahidin, I. Medicinal Uses, Phytochemistry and Pharmacology of Pongamia Pinnata (L.) Pierre: A Review. *J. Ethnopharmacol.* **2013**, *150*, 395–420.
16. Cecotti, R.; Carpana, E.; Bergomi, P.; Tava, A. Volatile Constituents of Trifolium Pratense Spp. Nivale Quantified at Different Growth Stages, and Evaluation of Their Antimicrobial Activity. *Nat. Prod. Commun.* **2013**, *8*, 1934578X1300801131.
17. Ramírez-López, C.B.; Beltrán-Sánchez, R.; Hernández-Izquierdo, A.; Salvadorhernández, J.L.; Salcedo-Pérez, E.; Del Río, R.E.; Pacheco, M.M.M. Antifeedant Activity of Caesalpinia Coriaria Essential Oil against Incisitermes Marginipennis (Latreille). *Phyton (B. Aires).* **2021**, *90*, 907.
18. Seigler, D.S. Phytochemistry of Acacia—Sensu Lato. *Biochem. Syst. Ecol.* **2003**, *31*, 845–873.
19. Van Den Boom, C.E.M.; Van Beek, T.A.; Posthumus, M.A.; De Groot, A.; Dicke, M. Qualitative and Quantitative Variation among Volatile Profiles Induced by Tetranychus Urticae Feeding on Plants from Various Families. *J. Chem. Ecol.* **2004**, *30*, 69–89.
20. da Silva, J.J.M.; Casoti, R.; Figueiredo, S.A.; Fonseca, M.J.V.; Rogez, H.; Heleno, V.C.G.; Bastos, J.K. Determination of the Composition of Copaifera (Fabaceae) Leaf Extracts with Potential Antioxidant Activity by Metabolomics Approach. *Rev. Bras. Farmacogn.* **2021**, 1–6.
21. Upadhyay, H.C.; Sisodia, B.S.; Cheema, H.S.; Agrawal, J.; Pal, A.; Darokar, M.P.; Srivastava, S.K. Novel Antiplasmodial Agents from Christia Vespertilionis. *Nat. Prod. Commun.* **2013**, *8*, 1934578X1300801123.
22. Falara, V.; Alba, J.M.; Kant, M.R.; Schuurink, R.C.; Pichersky, E. Geranyllinalool Synthases in Solanaceae and Other Angiosperms Constitute an Ancient Branch of Diterpene Synthases Involved in the Synthesis of Defensive

Compounds. *Plant Physiol.* **2014**, *166*, 428–441.
23. Caparrotta, S.; Comparini, D.; Marone, E.; Kimmenfield, R.; Luzzietti, L.; Taiti, C.; Mancuso, S. Correlation between VOC Fingerprinting and Antimicrobial Activity of Several Essential Oils Extracted by Plant Resins against A. Tumefaciens and P. Savastanoi. *Flavour Fragr. J.* **2019**, *34*, 377–387.
24. Jaeger, D.; O'Leary, M.C.; Weinstein, P.; Møller, B.L.; Semple, S.J. Phytochemistry and Bioactivity of Acacia Sensu Stricto (Fabaceae: Mimosoideae). *Phytochem. Rev.* **2019**, *18*, 129–172.
25. Rizwan, K.; Majeed, I.; Bilal, M.; Rasheed, T.; Shakeel, A.; Iqbal, S. Phytochemistry and Diverse Pharmacology of Genus Mimosa: A Review. *Biomolecules* **2022**, *12*, 83.
26. Tava, A.; Biazzi, E.; Ronga, D.; Pecetti, L.; Avato, P. Biologically Active Compounds from Forage Plants. *Phytochem. Rev.* **2022**, 1–31.
